# A Survey of Barley PIP Aquaporin Ionic Conductance Reveals Ca^2+^-Sensitive *HvPIP2;8* Na^+^ and K^+^ Conductance

**DOI:** 10.3390/ijms21197135

**Published:** 2020-09-27

**Authors:** Sen Thi Huong Tran, Tomoaki Horie, Shahin Imran, Jiaen Qiu, Samantha McGaughey, Caitlin S. Byrt, Stephen D. Tyerman, Maki Katsuhara

**Affiliations:** 1 Institute of Plant Science and Resources, Okayama University, 2-20-1 Chuo, Kurashiki 710-0046, Japan; pota42or@s.okayama-u.ac.jp (S.T.H.T.); ptj87a5q@s.okayama-u.ac.jp (S.I.); 2 Faculty of Agronomy, University of Agriculture and Forestry, Hue University, Hue 530000, Vietnam; 3 Division of Applied Biology, Faculty of Textile Science and Technology, Shinshu University, 3-15-1, Tokida, Ueda, Nagano 386-8567, Japan; horie@shinshu-u.ac.jp; 4 Australian Research Council Centre of Excellence in Plant Energy Biology, Waite Research Institute and School of Agriculture, Food and Wine, The University of Adelaide, Glen Osmond, Adelaide 5064, Australia; jiaen.qiu@adelaide.edu.au (J.Q.); Caitlin.Byrt@anu.edu.au (C.S.B.); steve.tyerman@adelaide.edu.au (S.D.T.); 5 Research School of Biology, Australian National University, Canberra 2600, Australia; Samantha.McGaughey@anu.edu.au

**Keywords:** aquaporins, barley, ion transport, oocytes, plasma membrane intrinsic proteins (PIPs)

## Abstract

Some plasma membrane intrinsic protein (PIP) aquaporins can facilitate ion transport. Here we report that one of the 12 barley PIPs (PIP1 and PIP2) tested, *HvPIP2;8*, facilitated cation transport when expressed in *Xenopus laevis* oocytes. *HvPIP2;8*-associated ion currents were detected with Na^+^ and K^+^, but not Cs^+^, Rb^+^, or Li^+^, and was inhibited by Ba^2+^, Ca^2+^, and Cd^2+^ and to a lesser extent Mg^2+^, which also interacted with Ca^2+^. Currents were reduced in the presence of K^+^, Cs^+^, Rb^+^, or Li^+^ relative to Na^+^ alone. Five HvPIP1 isoforms co-expressed with *HvPIP2;8* inhibited the ion conductance relative to *HvPIP2;8* alone but HvPIP1;3 and HvPIP1;4 with *HvPIP2;8* maintained the ion conductance at a lower level. *HvPIP2;8* water permeability was similar to that of a C-terminal phosphorylation mimic mutant *HvPIP2;8* S285D, but *HvPIP2;8* S285D showed a negative linear correlation between water permeability and ion conductance that was modified by a kinase inhibitor treatment. *HvPIP2;8* transcript abundance increased in barley shoot tissues following salt treatments in a salt-tolerant cultivar Haruna-Nijo, but not in salt-sensitive I743. There is potential for *HvPIP2;8* to be involved in barley salt-stress responses, and *HvPIP2;8* could facilitate both water and Na^+^/K^+^ transport activity, depending on the phosphorylation status.

## 1. Introduction

Aquaporins are well known for their transport of water and other small neutral solutes [1]. Higher plants have five aquaporin subfamilies (plasma membrane intrinsic proteins (PIPs), tonoplast intrinsic proteins (TIPs), Nodulin 26-like intrinsic proteins (NIPs), small basic intrinsic proteins (SIPs), and X-intrinsic proteins (XIPs) [2], where the PIP group aquaporins consist of two separate groups, PIP1 and PIP2. PIPs can influence plant hydraulic conductivity [3], and some have also been implicated in guard cell closure in response to ABA [4], in signaling in guard cells [5], and in CO_2_ sensing [6]. The regulation of PIPs in plants changes in response to salt treatments, and these changes might influence plant adaptation to salinity [7,8].

Salinity (NaCl) affects the expression of the PIP2 aquaporins in a time- and isoform-dependent manner [3,8]. Changes in aquaporin regulation in response to changes in salinity are particularly interesting because previous studies have revealed that a significant proportion of the highly abundant AtPIP2;1 protein relocates from the plasma membrane in *Arabidopsis* roots to an internalized location, and this could account for the reduction in root hydraulic conductance that is observed under salinity [9]. Targeting of PIPs to the plasma membrane and regulation of their internalization under salinity is dependent on the phosphorylation status of a serine (S283) in the carboxyl terminal domain [1,9].

Anion and cation transport properties have been reported for subsets of plant PIPs when expressed and tested in heterologous systems. For example, the rice (*Oryza sativa*) OsPIP1;3, which is upregulated in roots under drought stress, has recently been shown to be able to transport nitrate anions when expressed in mammalian HEK293 cells, and to also function as a water channel [10]. This aquaporin may be orthologous to the animal AQP6 that shows anion transport activity, though normally not water channel activity [11]. Two *Arabidopsis* PIPs, AtPIP2;1 and AtPIP2;2, have been shown to display non-selective cation conductance (K^+^ > Na^+^) when expressed in *Xenopus laevis* oocytes and these aquaporins have similar but not identical features to a human HsAQP1 ion and water channel aquaporin [12,13,14]. It was hypothesized that AtPIP2;1 could account for the voltage-independent non-selective cation channels (viNSCC) in plants [12] since the PIP2;1 and PIP2;2 cation conductance was inhibited by a low pH and by divalent cations [12,13], similar to the features observed for the viNSCCs observed in patch-clamp measurements on root protoplasts and roots [15,16,17]. There is also the similarity of inhibition of NSCCs and AtPIP2;1/AtPIP2;2 by cGMP [14,18]. There are other features of voltage-independent NSCCs that remain to be tested on the ion conducting PIP2s, including the selectivity to different monovalent cations. It is also unknown how many of the PIP2 isoforms of a species may induce ion conductance.

PIP1 and PIP2 isoforms can interact to form heterotetramers [19]. This interaction influences the movement of the PIP1 members to the plasma membrane [19,20], which also influences water transport, substrate selectivity, and pH dependence compared to the PIP2 homotetramers [12,21,22]. The cation transporting AtPIP2;1 was reported to have decreased cation transport when co-expressed with AtPIP1;2 in *X. laevis* oocytes [12], and this also resulted in increased water transport, as has been reported previously for several PIP1/PIP2 combinations [20,23,24].

Recently, the impact of phosphorylation at two C-terminal serines of AtPIP2;1, which are known to be differentially phosphorylated in *Arabidopsis* in response to changes in the environment [9], were examined in relation to water and cation transport characteristics [14]. This was undertaken since salinity stress results in changes in phosphorylation and membrane targeting of AtPIP2;1 [9,25,26,27]. When expressed in *X. laevis* oocytes, it was found that the phosphorylation status of S280 and S283 inferred from the phosphomimic mutation (serine to aspartic acid) or phospho-null mutation (serine to alanine) caused a reciprocal change in water and ion permeation. High ion conductance and low water permeability was more often associated with the DD mutation (S280D, S283D), while high water permeation and low ion conductance was associated with the AA mutation [14].

Barley (*Hordeum vulgare*) is an important grain crop worldwide and is relatively salt tolerant compared to other crops and *Arabidopsis* [28,29]. In barley, the analysis of an expressed sequence tag (EST) database and subsequent cDNA cloning have led to the identification of five *HvPIP1* and seven *HvPIP2* genes [24,30,31]. More recently, four more *HvPIP2* genes were identified by a search for barley aquaporin sequences on publicly available databases [32]. Robust water transport activity via HvPIP2;1 to HvPIP2;5 and *HvPIP2;8* have been demonstrated using *Xenopus laevis* oocytes [24,31]. HvPIP1;3 showed a relatively weak water transport activity, and all the other HvPIP1s showed no water channel activity when expressed alone in oocytes [24]. HvPIPs can influence the root hydraulic conductivity of barley, and the phosphorylation status and membrane internalization of the HvPIPs are implicated in the response of barley roots to salinity/osmotic stress [24,33,34,35,36]. Previous studies have revealed that there are multiple transporters influencing plasma membrane monovalent cation conductance in barley [37,38]. Hence, it is possible that the barley PIP2s could contribute to previously observed monovalent cation conductance across the barley plasma membranes, but this remains untested. Here, the barley PIP2s (from HvPIP2;1 to *HvPIP2;8* except HvPIP2;6) and PIP1s (HvPIP1;1 to HvPIP1;5) are surveyed to test for ion transport activity when expressed in *X. laevis* oocytes by two electrode voltage clamp (TEVC) experiments. We provide evidence that *HvPIP2;8*, an abundantly expressed aquaporin that shows water channel activity [31], also showed cation conductance. The *HvPIP2;8* cation selectivity, divalent cation sensitivity, interaction with PIP1 isoforms, and potential effects of differential phosphorylation states were examined, as well as the *HvPIP2;8* expression patterns in different barley cultivars.

## 2. Results

### 2.1. Ion Transport Activity Was Observed for HvPIP2;8 in Tests Screening for Barley PIP Ionic Conductance

**Figure 1:**
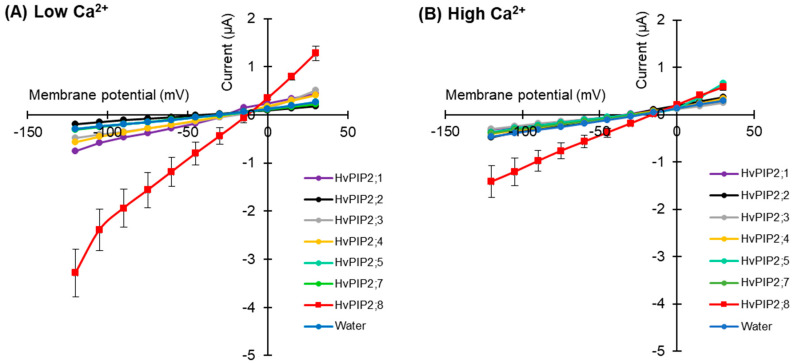

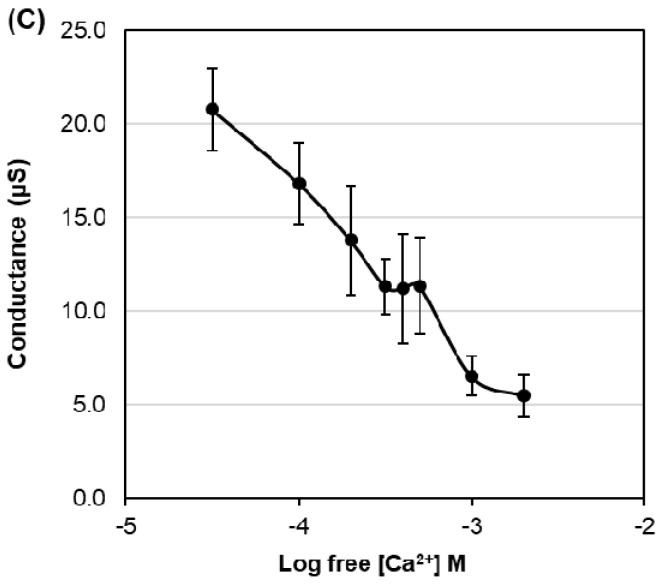
Electrophysiological survey to test for HvPIP2 ion transport (**A**,**B**) Current–voltage relationships of *X. leavis* oocytes expressing each *HvPIP2* in the presence of 86.4 mM NaCl and 9.6 mM KCl with 30 µM Ca^2+^ (**A**) or 1.8 mM Ca^2+^ (**B**). A total of 10 ng of each *HvPIP2* cRNA or water (control) was injected into *X. laevis* oocytes. (**C**) Relationships between the external free Ca^2+^ concentration and *HvPIP2;8*-mediated Na^+^ conductance in the presence of 86.4 mM NaCl and 9.6 mM KCl (R^2^ = 0.93). The free Ca^2+^ concentrations are given in Methods. A step pulse protocol of −120 mV to +30 mV with a 15 mV increment was applied on every oocyte. Ionic conductance was calculated based on the data obtained from V = −75 mV to −120 mV of the membrane potential. Data are the means ± SE (n = 5 for **A**,**C**, and n = 7 for **B**).

To test for HvPIP ion transport activity, two electrode voltage clamp (TEVC) experiments were conducted using *X. laevis* oocytes expressing HvPIP2s (from HvPIP2;1 to *HvPIP2;8*). This revealed that only the expression of *HvPIP2;8* elicited large bidirectional and voltage-independent currents in the bath solution, including 86.4 mM NaCl and 9.6 mM KCl, and 30 µM free Ca^2+^ (low Ca^2+^ condition) (Figure 1A). Small currents were observed for HvPIP2;1 in low Ca^2+^ conditions, and in some experiments these currents were greater than the currents recorded for the water-injected controls (Table S2). When the bath solution contained a high Ca^2+^ concentration, 1.8 mM Ca^2+^, the *HvPIP2;8*-associated currents were smaller than in low Ca^2+^ conditions (Figure 1B). The *HvPIP2;8*-associated ionic conductance was 36.46 and 14.50 µS in low and high external free Ca^2+^ conditions, respectively, and these values were significantly higher than the ionic conductance of the water-injected oocytes (3.31 and 3.44 µS, respectively: Supplementary Table S2). HvPIP2;2, HvPIP2;3, HvPIP2;4, HvPIP2;5, and HvPIP2;7 did not elicit ionic conductance that was significantly different to that of the water-injected oocytes (Figure 1A,B, Supplementary Table S2).

The ionic conductance induced by *HvPIP2;8* was further examined in response to various concentrations of Ca^2+^ supplemented in a 86.4 mM NaCl and 9.6 mM KCl bath solution. Interestingly, the ionic conductance was strongly inhibited in accordance with increases in the external free Ca^2+^ concentration (Figure 1C, Supplementary Figure S1). This result suggested a negative correlation between the *HvPIP2;8*-mediated ionic conductance and the external free Ca^2+^ concentrations. In all experiments, no shift of the reversal potential was observed at high or low external calcium concentrations (Supplementary Table S3), indicating that the channel was not permeable to Ca^2+^.

### 2.2. HvPIP2;8 Monovalent Alkaline Cation Selectivity

**Figure 2:**
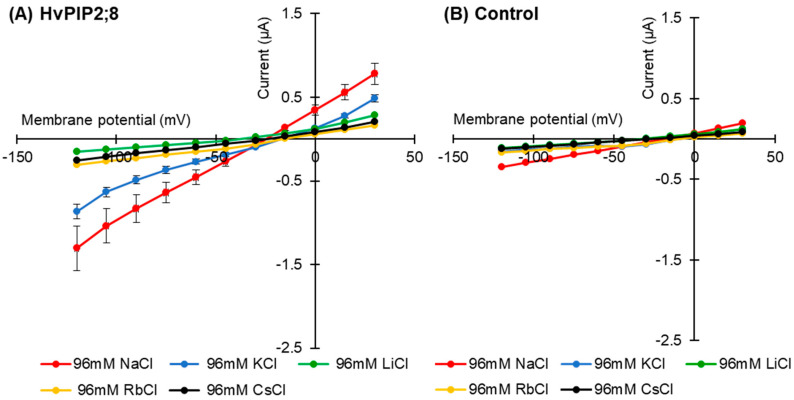

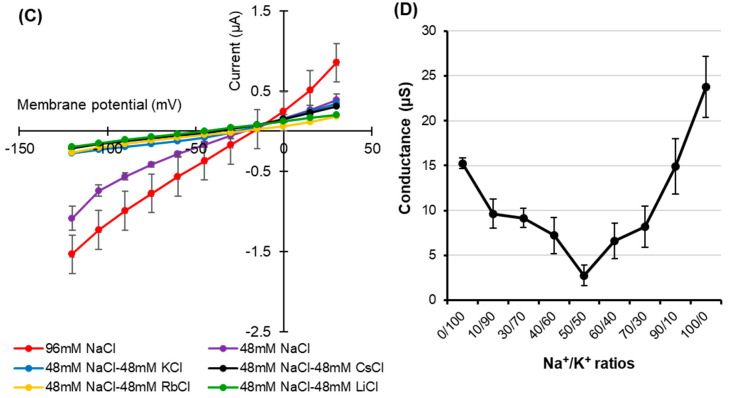
Monovalent alkaline cation selectivity of *HvPIP2;8* and the effect of the interaction of K^+^ and Na^+^ on *HvPIP2;8*-mediated ion conductance activity. Current–voltage relationships obtained from oocytes either expressing *HvPIP2;8* (**A**) or injected with water (**B**) *HvPIP2;8* displays a different monovalent alkaline cation selectivity. Oocytes were successively immersed in bath solutions with a high calcium condition, supplemented with Na^+^, K^+^, Cs^+^, Rb^+^, and Li^+^ (as chloride salts) at the concentration of 96 mM. (**C**) Inhibition of *HvPIP2;8*-mediated Na^+^ transport by monovalent alkaline cations in the presence of 48 mM NaCl with 48 mM of each alkaline cation. (**D**) The effect of external Na^+^/K^+^ concentration ratios on the conductance of *HvPIP2;8*-expressing oocytes from V (membrane potential) = −75 mV to −120 mV. The total concentration of (Na + K) was constantly 96 mM. *X. laevis* oocytes were injected with 10 ng of *HvPIP2;8* cRNA for the recording of the conductance in every experiment. Data are the means ± SE (n = 7 to 8 for **A**, n = 5 for **B**, n = 4 to 5 for **C**, and n = 5 to 6 for **D**).

Current–voltage relationships for the *HvPIP2;8*-expressing oocytes were recorded in the presence of 96 mM Na^+^, K^+^, Rb^+^, Cs^+^, or Li^+^ (as chloride salt; Figure 2A). When the oocytes were bathed in a 96 mM Li^+^, Rb^+^, or Cs^+^ solution, the *HvPIP2;8*-associated currents did not differ from the background currents recorded for the water-injected control oocytes; ionic conductance was only detected for the *HvPIP2;8*-expressing oocytes when bathed in either a Na^+^- or K^+^-containing solution (Figure 2A,B).

*HvPIP2;8*-associated currents were then measured in the presence of solutions with combinations of different monovalent cations. Current–voltage relationships were obtained from *HvPIP2;8* expressed in oocytes bathing in 48 mM Na^+^ solutions in the co-presence of either 48 mM K^+^, Cs^+^, Rb^+^, or Li^+^ (as chloride salt). Smaller *HvPIP2;8*-associated currents were observed in Na^+^ solutions when other monovalent cations were added to the solution (Figure 2C). A positive shift in the reversal potential was observed in 96 mM NaCl solutions relative to 48 mM NaCl solutions, consistent with *HvPIP2;8* mediating Na^+^ transport (Figure 2C, Supplementary Figure S2). However, in the presence of solutions containing 48 mM KCl and 48 mM NaCl, there were smaller *HvPIP2;8*-mediated Na^+^ currents than in solutions with only 48 mM NaCl (Figure 2C). The use of solutions that included different combinations of Na^+^ and K^+^ concentrations revealed that an external Na^+^:K^+^ ratio of 50:50 limited the ionic conductance of the *HvPIP2;8*-expressing oocytes; the magnitude of the currents in the 50:50 ratio solutions was 88.4% and 81.9% of the magnitude of the currents in a 100:0 or 0:100 Na^+^:K^+^ ratio solution, respectively (Figure 2D, Supplementary Figure S3). These observations indicated that the Na^+^ permeability of *HvPIP2;8* appears to be highly dependent on the external K^+^ concentration.

### 2.3. HvPIP2;8 Was not Permeable to Cl^−^

**Figure 3:**
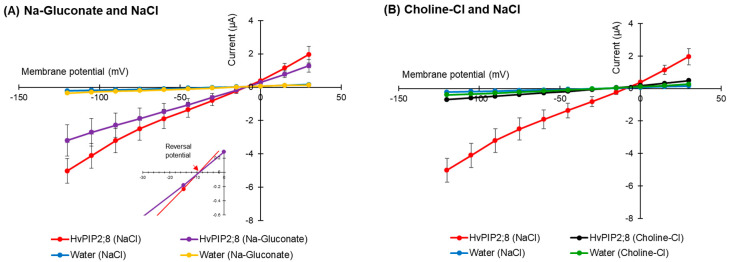
*HvPIP2;8*-mediated Na^+^ transport is Cl^−^ independent. (**A**) Current–voltage relationships obtained from oocytes either expressing *HvPIP2;8* or injected with water in the presence of either 96 mM NaCl or 96 mM Na-gluconate. Inset: Expanded current voltage curves around the reversal potential. (**B**) Current–voltage relationships obtained from oocytes either expressing *HvPIP2;8* or injected with water in the presence of either 96 mM NaCl or 96 mM Choline-Cl. All solutions contained 30 µM Ca^2+^. *X. laevis* oocytes were injected with 10 ng of *HvPIP2;8* cRNA. Data are the means ± SE (n = 7 to 8).

The effect of the presence of the external anion Cl^−^ on *HvPIP2;8* ion transport was tested using Na-gluconate and Choline-Cl solutions (96 mM each). Similar current–voltage relationships for *HvPIP2;8*-expressing oocytes were observed regardless of whether there was Cl^−^ or gluconate solutions used in the bath (Figure 3A), and there was no shift in the reversal potential (−9 mV) for the different solutions, indicating that the *HvPIP2;8*-induced currents were not affected by Cl^−^. In the presence of 96 mM Choline-Cl, the *HvPIP2;8*-expressing oocytes elicited minor currents (−0.68 ± 0.42 µA at −120 mV), comparable to those of the water-injected control oocytes (−0.39 ± 0.03 µA at −120 mV), which was significantly different to the currents observed in the presence of 96 mM NaCl (Figure 3B). These results indicated that the *HvPIP2;8*-associated Na^+^-induced currents across the plasma membrane of the oocytes were not affected by the external Cl^−^ concentration.

### 2.4. Effects of Divalent Cations on HvPIP2;8-Mediated Ion Transport Activity

**Figure 4:**
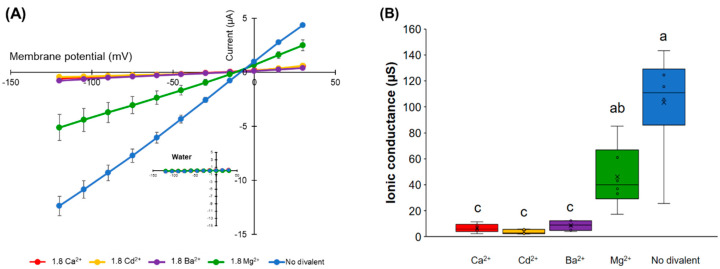

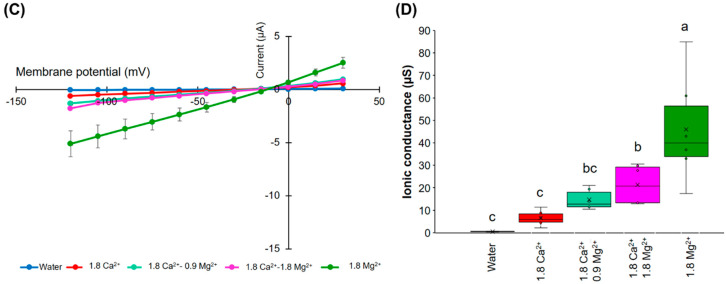
Effects of divalent cations on the ion current responses in oocytes expressing *HvPIP2;8*. (**A**) Effect of divalent cations on the ion currents of the *HvPIP2;8*-transporter; bath solutions with a high 1.8 mM Ca^2+^ background calcium conditions were successively replaced with either 1.8 mM Ca^2+^, Ba^2+^, Cd^2+^, and Mg^2+^ (as chloride salts), at concentrations of 86.4 mM NaCl and 9.6 mM KCl. (**B**) Box plot summary of the ionic conductance presented in (**A**); the ionic conductances were calculated from V = −75 mV to −120 mV. (**C**) Relief of Ca^2+^ inhibition by the addition of Mg^2+^ on the ion current responses in oocytes expressing *HvPIP2;8*; note the different range of the *Y*-axis from the plot in (**A**). (**D**) Box plot summary of the ionic conductance presented in (**C**). Steady-state current–voltage curves of the *X. laevis* oocytes injected with 10 ng of cRNA per oocyte were recorded. Currents from the oocytes injected with water were the negative controls from the same batch. Significant differences (*p* < 0.05) are indicated by different letters using one-way ANOVA with Duncan’s multiple comparisons test. Data are the means ± SE of three independent experiments, (n = 6 for **A**,**B**).

The effect of different divalent cations on the ion conductance activity of *HvPIP2;8* was tested. Maximal ionic conductance associated with *HvPIP2;8* was observed when the oocytes were bathed in a divalent cation-free saline (86.4 mM NaCl, 9.6 mM KCl, 10 mM HEPES, pH 7.5 with Tris, and osmolality was adjusted to 200 mosmol Kg^−1^ with supplemental mannitol). However, the *HvPIP2;8* channel was inhibited by the extracellular application of 1.8 mM Ba^2+^, Cd^2+^, or Ca^2+^ (Figure 4A). In contrast, the application of 1.8 mM MgCl_2_ gave rise to a weaker inhibitory effect on the *HvPIP2;8*-mediated ion currents (Figure 4A,B).

An increase in the MgCl_2_ concentration in the presence of 1.8 mM CaCl_2_ tended to partially cancel out the inhibitory effect of Ca^2+^ in relation to the ion channel activity (Figure 4C,D). These results indicate that there may be a competitive interaction between Ca^2+^ and Mg^2+^, which influences *HvPIP2;8*-mediated ion channel activity and the presence of more external Mg^2+^ can partially relieve the inhibitory effect of high Ca^2+^ on *HvPIP2;8* ionic conductance.

### 2.5. Co-Expression of HvPIP2;8 with HvPIP1s Limited HvPIP2;8 Ion Transport Activity

**Figure 5:**
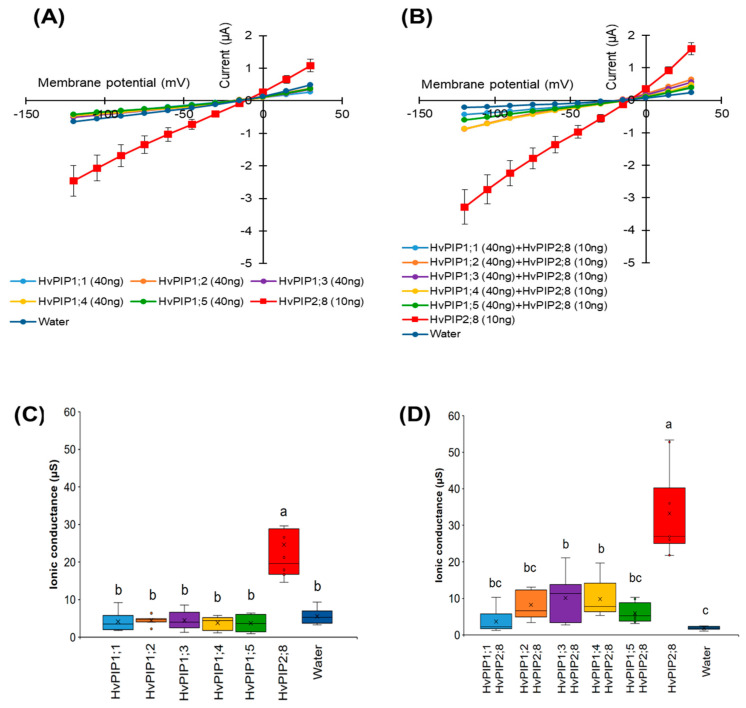
Co-expression of HvPIP1s and *HvPIP2;8* reduces *HvPIP2;8*-dependent currents in *X. laevis* oocytes. (**A**) Current–voltage relationships obtained from oocytes either expressing *HvPIP2;8* alone, each HvPIP1 alone, or injected with water; each HvPIP1 of the five HvPIP1s did not show ion channel activity when expressed alone. (**B**) Co-expression of *HvPIP2;8* with each HvPIP1 largely inhibited the ion channel activity of *HvPIP2;8*. (**C**,**D**) Box plot summary of the ionic conductance for data shown in (**A**,**B**), respectively. Oocytes were injected with 10 ng cRNA of *HvPIP2;8*, 40 ng cRNA of each HvPIP1 in both the solo- and co-expression analyses. The bath solution included 86.4 mM NaCl, 9.6 mM KCl, 1.8 mM MgCl_2_, 1.8 mM EGTA, 1.8 mM CaCl_2_, 10 mM HEPES, and a pH 7.5 with Tris, and therefore the free Ca^2+^ concentration was 30 µM. Significant differences (*p* < 0.05) are indicated by different letters using one-way ANOVA with Duncan’s multiple comparisons test. Data are the means ± SE (n = 8).

Co-expression of HvPIP1;2 and HvPIP2;1-2;5 resulted in increases in water transport across the plasma membrane of the oocytes, relative to the water transport of oocytes expressing HvPIP2s alone [24], but *HvPIP2;8* was not included in that work [31]. Here, we examined the effect of the co-expression of HvPIP1s (HvPIP1;1 to HvPIP1;5) with *HvPIP2;8* in relation to ionic conductance. When expressed alone, each HvPIP1 displayed similar currents to the water-injected controls (Figure 5A,C). However, when each HvPIP1 was co-expressed with *HvPIP2;8*, the large *HvPIP2;8*-associated currents were not observed in any of the co-expression combinations examined, whereas when *HvPIP2;8* was expressed alone, the *HvPIP2;8*-associated currents were significant as expected (Figure 5B,D). These results indicate that HvPIP1s might be interacting with *HvPIP2;8* and that this either prevents, or significantly reduces (for HvPIP1;3 and HvPIP1;4), the *HvPIP2;8* ion channel activity.

### 2.6. An HvPIP2;8 S285D Phosphomimic Mutant Had Greater Ionic Conductance Than Wild Type HvPIP2;8

**Figure 6:**
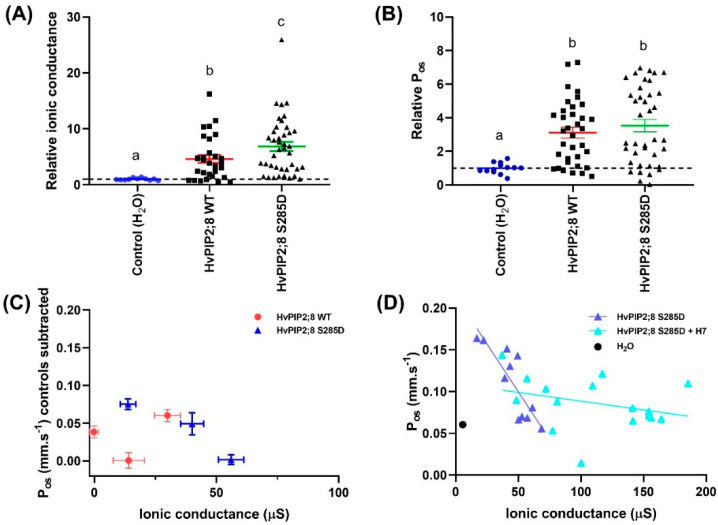
Phosphorylation mimic *HvPIP2;8* S285D influences *HvPIP2;8*-facilitated cation transport. Oocytes were injected with 46 nL water (Control) or with 46 nL water (n = 11) containing 23 ng *HvPIP2;8* WT (n = 30) or *HvPIP2;8* S285D (n = 40) cRNA. Ionic conductance and osmotic water permeability (P_os_) of the cRNA-injected oocytes were determined via the TEVC and the swelling assay, respectively. (**A**) Na^+^ conductance relative to H_2_O-injected control (dotted line). Currents were recorded in “Na100” (100 mM NaCl; 2 mM KCl, 1 mM MgCl_2_, 5 mM HEPES, 50 μM CaCl_2_, 100 mM NaCl or 100 mM KCl, osmolality 220 mosmol Kg^−1^ (adjusted with D-mannitol), and pH 8.5). (**B**) P_os_ relative to H_2_O-injected control (dotted line). Data in (**A**,**B**) was collected from three different frogs and is shown as the mean ± SE, where each data point represents an individual oocyte; for each oocyte, both the ionic conductance and P_os_ were measured; data from (**C**,**D**) is from one batch and again for each oocyte both the ionic conductance and P_os_ were measured. Significant differences (*p* < 0.05) are indicated by different letters (one-way ANOVA, Fisher’s post-test). (**C**) Relationships between the mean P_os_ and mean ionic conductance for *HvPIP2;8* WT and *HvPIP2;8* S285D with the mean of the controls subtracted. This data is from oocytes from the same three independent frogs as shown in (**A**,**B**). (**D**) Kinase inhibitor H7 influenced the relationship between the ionic conductance and water permeability in *HvPIP2;8* S285D-expressing oocytes. Oocytes were either untreated or were pre-treated in a low Na^+^ Ringer solution that contained 10 µM H7 dihydrochloride (H7) for 2 h before TEVC and the swelling assay. An individual conductance was plotted against the corresponding P_os_ for each oocyte, and the mean for the water-injected controls is shown (black circle, dotted line). Linear regression of P_os_ versus ionic conductance was only significant for *HvPIP2;8* S285D without H7 treatment (*p* < 0.005).

To investigate the possibility of a regulatory role for a C-terminal tail serine at residue 285, a phosphomimic mutant of *HvPIP2;8* was generated where the site coding for this residue was mutated such that it coded for aspartic acid (D) instead of serine (S). The ionic conductance and osmotic water permeability (P_os_) of this mutant was compared to the wild type *HvPIP2;8* and water-injected controls in oocytes from three independent frogs. The ionic conductance observed for *HvPIP2;8* S285D was greater than the ionic conductance for *HvPIP2;8* and nearly seven-fold higher than the water-injected controls (Figure 6A; Supplementary Figure S4A). There was no significant difference in the mean P_os_ for *HvPIP2;8* S285D relative to *HvPIP2;8* WT, but there was a 3.5-fold variability in the magnitude of P_os_ as well as variability between batches (Figure 6B,C; Supplementary Figure S4B). To explore whether phosphorylation at *HvPIP2;8* S285 could influence the P_os_ and ionic conductance relationship, simple linear regressions were fitted for the *HvPIP2;8* wild type and *HvPIP2;8* S285D between the P_os_ and ionic conductance from oocytes harvested from three independent frogs (Figure 6C). To remove the frog batch variability in the P_os_ and ionic conductance, the mean of P_os_ and ionic conductance of the H_2_O controls were subtracted from both the *HvPIP2;8* wild type and *HvPIP2;8* S285D (Figure 6C). No significant relationship between the P_os_ and ionic conductance was observed for the wild type *HvPIP2;8*, whereas for the *HvPIP2;8* S285D-expressing oocytes, a negative reciprocal relationship was observed (Figure 6C,D).

We investigated whether the activity of the endogenous kinases in the *X. laevis* oocytes might influence the phosphorylation state of *HvPIP2;8* or *HvPIP2;8* S285D by applying a kinase inhibitor H7. The H7 treatment significantly increased the ionic conductance and reduced the P_os_ of *HvPIP2;8* S285D relative to the *HvPIP2;8* S285D-expressing oocytes that were not subjected to the H7 treatment (Figure 6D, Supplementary Figure S4C). Analysis of the predicted phosphorylation sites in *HvPIP2;8* (http://www.cbs.dtu.dk/services/NetPhos/) indicated that there are nine candidate sites targeted by protein kinase A or C, which are the kinases involved in *Xenopus* oocyte signal transduction pathways. S285 was not included in the nine candidate sites predicted by NetPhos, indicating that the endogenous oocyte kinases are likely to target an alternative site or sites ( Supplementary Figure S4D,E; [39]).

### 2.7. Expression of HvPIP2;8 in Barley

**Figure 7:**
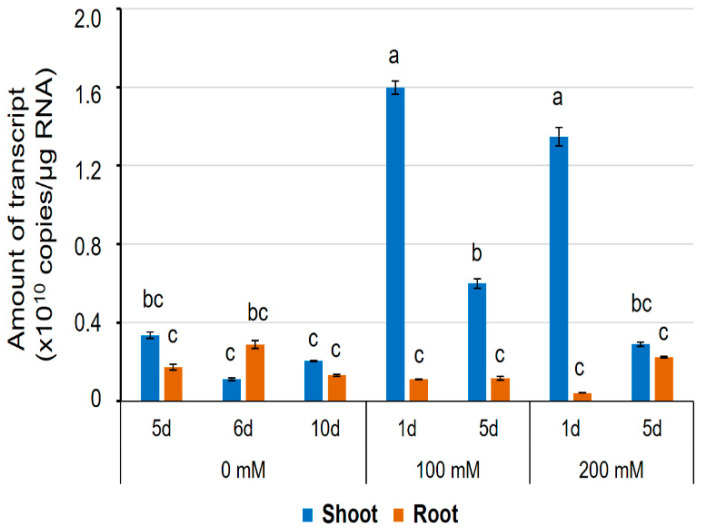
The expression level of the *HvPIP2;8* transcripts in a barley cultivar, Haruna-Nijo, detected by qPCR. Five-day-old barley seedlings were prepared by hydroponic culture and further grown on the culture solution with or without NaCl (100 mM or 200 mM) for 1 day or 5 days. Transcript levels of *HvPIP2;8* in shoots and roots were investigated by absolute quantification. Absolute amounts of transcripts (copies/µg RNA) were displayed. Significant differences (*p* < 0.05) are indicated by different letters using one-way ANOVA with Duncan’s multiple comparisons test. Data are the means ± SE, and n = 3.

Previously, *HvPIP2;8* was observed to be stably expressed in shoots, roots, pistils, and leaves [31]. In this study, to further explore the transcript regulation of *HvPIP2;8*, qPCR was used to assess expression in salt-treated and control shoot and root samples from the barley cultivar Haruna-Nijo (Figure 7). In roots, the transcript levels remained stable in both the salt-treated and control samples (Figure 7). However, in shoots from Haruna-Nijo plants, *HvPIP2;8* transcripts were more abundant in the salt-treated samples than the control samples after 1 day of either the 100 mM or 200 mM NaCl treatment. After five days of NaCl treatment, the *HvPIP2;8* transcripts were less abundant in the shoots than they were at 1 day after NaCl treatment (Figure 7). RT-PCR showed that the transcript levels of *HvPIP2;8* were increased in shoots of the salt-tolerant cultivar, K305 subjected to a 200 mM NaCl treatment relative to the controls (Supplementary Figure S5). Whereas, in the salt-sensitive cultivar, I743, there was no difference in the abundance of *HvPIP2;8* in the salt- and control-treated samples (Supplementary Figure S5). This indicates that the *HvPIP2;8* gene expression in shoots of barley cultivars might vary depending on cultivar and environmental conditions.

## 3. Discussion

*HvPIP2;8* has the potential to function as both a water and a cation channel, where the channel characteristics can be influenced by K^+^ and divalent cation activity, protein phosphorylation, and protein interactions, and where the *HvPIP2;8* transcript levels can be influenced by salt treatments. We observed that *Xenopus* oocytes expressing *HvPIP2;8* displayed significant ionic conductance relative to the controls and relative to the oocytes expressing any of the six other HvPIP2s and five HvPIP1 proteins. Previous studies have demonstrated water channel activity for HvPIP2;1, HvPIP2;2, HvPIP2;3, HvPIP2;4, HvPIP2;5, and HvPIP2;7 when expressed in oocytes, indicating that the low or absent ionic conductance associated with expression of these proteins is unlikely to relate to miss-folding or miss-targeting [24,35].

*HvPIP2;8*-associated ionic conductance was inhibited by external Ca^2+^, Cd^2+^, and Ba^2+^, but less so by Mg^2+^ (Figure 4B). *HvPIP2;8* was permeable to both Na^+^ and K^+^, and the Na^+^ permeability of *HvPIP2;8* was inhibited in the presence of external K^+^, but not external Cl^−^. Co-expression of *HvPIP2;8* and HvPIP1 proteins reduced the *HvPIP2;8*-induced ionic conductance with differences between the PIP1 isoforms; HvPIP1;3 and HvPIP1;4 co-expressed with *HvPIP2;8* still maintained a higher ionic conductance than the water-injected controls (Figure 5D). Greater ionic conductance was observed for an S285D mutant version of *HvPIP2;8* relative to the wild type, but no difference in the P_os_. Treatments to manipulate oocyte kinase activity differentially influenced wild type *HvPIP2;8* relative to the S285D mutant. The salt-tolerant and salt-sensitive barley lines differed in their response to salt treatments, such that the salt tolerant cultivars tested displayed an increase in the abundance of *HvPIP2;8* transcripts within the first day after the salt treatments.

In saline conditions, excessive salt accumulation is detrimental to plant growth and limits crop productivity. This problem is often referred to as ionic toxicity, and for many cereals it is brought about by excessive Na^+^ influx into roots followed by excess Na^+^ accumulation, particularly in the aerial parts of the plants [40]. Uptake of Na^+^ at the root–soil boundary is conferred by multiple pathways involving a range of different types of membrane transporters and channels. For example, OsHKT2;1, one of the high-affinity K^+^ transporter family proteins in rice, mediates direct Na^+^ absorption from the outer environment of roots when the rice plant faces K^+^ starvation conditions [41]. The roles of some important Na^+^ transporters, such as the SOS1, NHX, and HKT families, which contribute to salt-stress resistance, have been well characterized [28]. However, some pathways for Na^+^ influx into plant roots remain unresolved at the molecular level, although we assume owing to electrophysiological studies that non-selective cation channels (NSCC) mediate significant Na^+^ influx into roots following salinity stress [15,16,17,37]. Candidates for NSCCs include cyclic nucleotide-gated channels (CNGCs) and glutamate receptors (GLRs), but confirmation of the molecular identity of the NSCC will require further research [42,43]. In the future, determining the structure and the role of unidentified Na^+^ permeable transporters/channels in plants that contribute to NSCC activity, including Na^+^ permeable aquaporins, will help us to understand the complete picture of Na^+^ transport and homeostasis during salinity stress.

Aquaporins are well known for their function as water channels [34,35,36,44]. Previous research has revealed that heterologous expression of AtPIP2;1, categorized as a plasma membrane-localized aquaporin in *Arabidopsis thaliana*, is associated with non-selective cation conductance, and this ion channel function is sensitive to Ca^2+^ [12]. More recently, the Ca^2+^ sensitivity of another water and ion channel aquaporin, AtPIP2;2, was revealed [13]. In the present study, we used TEVC experiments to screen *X. laevis* oocytes expressing barley HvPIPs and the controls, and this revealed significant *HvPIP2;8*-associated ion channel activity under low Ca^2+^ conditions. Among the set of 12 HvPIPs tested, *HvPIP2;8* stood out in relation to conferring ion channel activity in oocytes (Figure 1A and Figure 5A). The screen revealed that HvPIP2;1 might be capable of facilitating ionic conductance, but the HvPIP2;1-associated currents were significantly smaller than the *HvPIP2;8*-associated currents (Figure 1A, Supplementary Table S2). It is also noteworthy that all the plant aquaporins so far shown to conduct cations in the *Xenopus* oocytes show different characteristics in terms of cation selectivity and divalent cation inhibition. The human AQP1, which has an ion channel function, also shows differences in channel characteristics relative to PIP2;1 and PIP2;2 [13]. This would strongly suggest that the aquaporins are not triggering a native *Xenopus* channel to be activated or recruited to the membrane. The effect of the phospho-mimic mutations in *HvPIP2;8* and AtPIP2;1 discussed below is also difficult to explain in terms of recruitment of a native *Xenopus* channel.

As *HvPIP2;8* has been demonstrated to conduct water and ions when expressed in oocytes ([31]; Figure 1), we hypothesize that this aquaporin can function as a channel that mediates both water and ion transport in plants. The Na^+^ channel activity associated with *HvPIP2;8* was sensitive to external Ca^2+^ concentrations (Figure 1B,C), and the IC_50_ of the channel was calculated to be approximately 401 μM (https://www.aatbio.com/tools/ic50-calculator), which was similar to the Ca^2+^ sensitivity of the AtPIP2;1 ionic conductance (IC_50_ = 321 μM; [12]). The inhibition by Ca^2+^ is not total since even at 1.8 mM there is still a significant ion conductance (Figure 4D). The analysis of alkali monovalent cations selectivity revealed that *HvPIP2;8* mediated not only Na^+^ but also K^+^ transport, although *HvPIP2;8* did not mediate Rb^+^, Cs^+^, or Li^+^ transport (Figure 2A,B). However, these monovalent cations, and K^+^, blocked the Na^+^ channel activity of *HvPIP2;8* when the same amount of each cation was included in the bath solutions (Figure 2C,D). Inhibition or activation of Na^+^ transport activity by the presence of similar or greater concentrations of external K^+^ in the TEVC bath solution has been observed for different types of high-affinity K^+^ transport (HKT)-type sodium transporters. For example, *Triticum aestivum* TaHKT1;5-D and *Triticum monococcum* TmHKT1;5-A encode dual affinity Na^+^-transporters and their dual affinity Na^+^ transport was inhibited by raising the external K^+^ concentration [45,46]; whereas, for OsHKT2;2, extracellular K^+^ stimulated the OsHKT2;2-mediated Na^+^ transport [47]. Additional research is needed to model how different monovalent ions interact with the pore lining residues of the ion channel aquaporins towards understanding the K^+^ inhibition effect on the *HvPIP2;8* Na^+^ channel activity.

The influence of divalent cations on the *HvPIP2;8*-mediated Na^+^ currents was complicated. The application of 1.8 mM Ba^2+^, Ca^2+^, or Cd^2+^ inhibited the *HvPIP2;8* ionic conductance (Figure 4A,B). The application of the same amount of Mg^2+^ in the bath solution did not have an equivalent inhibitory influence on the ionic conductance (just an approximately 63% reduction compared to no divalent control: Figure 4A,B). However, an increase in the Mg^2+^ concentrations in the presence of Ca^2+^ seemed to ameliorate the inhibitory effect of the Ca^2+^ (Figure 4C,D). The competitive interaction between Ca^2+^ and Mg^2+^ might be due to a higher affinity for Mg^2+^ than for Ca^2+^. Alternatively, Mg^2+^ might interact with the same binding site as Ca^2+^. Previous research revealed that the AtPIP2;1 and AtPIP2;2 ionic conductance was significantly inhibited by 100 µM and 10 µM extracellular free Ca^2+^, respectively, and the ionic conductance was also significantly inhibited by the addition of Ba^2+^ and Cd^2+^ [13]. There was also an interaction between Ca^2+^ inhibition and Ba^2+^ relief of block for AtPIP2;1 [13] that is similar to the interaction seen here for Ca^2+^ and Mg^2+^. The determination of specific interaction sites for divalent cations in the structure of *HvPIP2;8* will be essential to understanding the characteristics observed.

A previous study showed that co-expression of *HvPIP2;8* with HvPIP1;2 in *X. laevis* oocytes did not enhance the water transport activity compared to that of the expression of *HvPIP2;8* alone [31]. In contrast, we observed that co-expression of HvPIP1;2 with other HvPIP2s (2;1 to 2;5) increased the water permeability coefficient [24]. This indicates that heteromerization of each HvPIP2 and HvPIP1;2 could modulate water channel activity differently. We observed here that co-expression of *HvPIP2;8* with the HvPIP1s, including HvPIP1;2, significantly decreased the ionic conductance relative to expression of *HvPIP2;8* alone (Figure 5), indicating that the *HvPIP2;8*-mediated ion channel activity might be negatively regulated through heteromerization. Some isoforms retained some ion conductance when co-expressed (HvPIP1;3 and HvPIP1;4). A previous study revealed that when AtPIP2;1 was co-expressed with AtPIP1;2 the water permeability was greater than when AtPIP2;1 or AtPIP1;2 was expressed alone [12]. However, the ionic conductance of AtPIP2;1 could be suppressed to the level of the water-injected controls when AtPIP2;1 was co-expressed with AtPIP1;2, indicating that the ionic conductance was not associated with higher water permeability [12]. However, this was done at high external Ca^2+^ concentration and it remains to be seen if the same result would be obtained at lower external Ca^2+^. Together, these results reveal that the activity for water and ion channel PIPs could be differently regulated by independent mechanisms. At present, the mechanism of HvPIP1-dependent decreases in the ion transport activity of *HvPIP2;8* is still unknown. It might be related to changes in the central tetrameric pore dimensions comparing the ion conducting homotetramer with the heterotetramer since the central tetrameric pore is the favored pathway for ion conductance through AQP1 [48]. Elucidating the underlying molecular mechanisms will be important to understand the functions of *HvPIP2;8* as a Na^+^-permeable ion channel.

The water and ion channel functions, and the membrane localization of the aquaporins, have previously been reported to be influenced by the phosphorylation status of the C-terminal domain (CTD). For example, versions of AtPIP2;1 mimicking either a phosphorylated state or unphosphorylated state of residues 280 and 283 by mutating these serine residues to either an aspartic acid (D) or an alanine (A) were used to test whether changes in phosphorylation in the CTD might influence AtPIP2;1 water and ion channel function [14]. This revealed that the phosphorylation mimic mutants S280D, S283D, and S280D/S283D had a significantly greater ion conductance for Na^+^ and K^+^, whereas the phosphonull mutants S280A single and S280A/S283A double had greater water permeability. Interestingly, among the HvPIP2s, *HvPIP2;8* is the only HvPIP2 lacking an AtPIP2;1 S280 equivalent site at CTD; and *HvPIP2;8* also lacks a serine on loop D that is present in AtPIP2;1 (Supplementary Figure S4F). As a first step towards exploring whether changes in phosphorylation of the *HvPIP2;8* CTD may influence water ion channel function, a phosphomimic mutant S285D was generated. The ionic conductance of *HvPIP2;8* S285D was greater than that of *HvPIP2;8* WT, but the P_os_ was similar (Figure 6A,B). Treatments with a kinase inhibitor, H7, resulted in an increase in S285D ionic conductance and a decrease in P_os_ relative to the untreated S285D (Figure 6D). H7 influences the activity of endogenous kinases, which can alter the phosphorylation state of the aquaporins that have been heterologously expressed in the oocytes [14,39,49]. The observation that treatment with H7 can increase the ionic conductance and decrease the P_os_ of *HvPIP2;8* S285D indicates that there is likely to be residues, other than S285, where differential phosphorylation of these additional residues in *HvPIP2;8* can influence the water and ion channel activity. A significant negative relationship between P_os_ and ionic conductance was observed for *HvPIP2;8* S285D (Figure 6D), suggesting a mutually exclusive gating of ion and water flow for this mutant, which was also observed for the AtPIP2;1 phospho-mimic mutants [14]. There are nine *HvPIP2;8* residues that are candidate sites for potentially being phosphorylated by the endogenous oocyte kinases PKA and PKC (Supplementary Figure S4D,E), including a serine site on loop D that could be influential in gating based on previous structural analysis of *Spinacia oleracea* SoPIP2;1 [50]. The next step towards determining which additional sites are targeted by endogenous oocyte kinases will be require testing of additional mutant versions of *HvPIP2;8*; this will assist in determining which phosphorylation sites may be part of the post-translational mechanisms for regulating *HvPIP2;8* water and ion channel function.

In barley, the *HvPIP2;8* gene expresses in both roots and shoots (Figure 7). Interestingly, *HvPIP2;8* expression in shoots was upregulated in response to salt stress (Figure 7). An RT-PCR analysis revealed that the upregulation trend for *HvPIP2;8* transcript abundance was observed in salt-tolerant barley, but not detected in a salt-sensitive barley cultivar (Supplementary Figure S5). These observations, and the characteristics of the *HvPIP2;8* observed by TEVC experiments, led us to wonder whether *HvPIP2;8* could play a positive role in shoot tissues to help cope with salt stress. We observed that the external free Ca^2+^ concentrations have a significant impact on the ion channel activity of *HvPIP2;8* (Figure 1 and Figure 5, and Supplementary Figure S1). It is well known that Ca^2+^ plays key roles in ameliorating Na^+^ toxicity under salt stress [51]. In addition, changes in free Ca^2+^ have important signaling roles, particularly in response to stress conditions [52,53]. The implications of Ca^2+^ sensitivity could be different depending on what kind of physiological role *HvPIP2;8* has in planta: for example, if the *HvPIP2;8* mediated the Na^+^ influx into the cytosol of mesophyll cells in leaves, then a Ca^2+^-dependent inhibitory effect might be a positive feature as it could prevent excess Na^+^ influx; or if *HvPIP2;8* played a role analogous to the role of the HKT1s, some of which are known to function in unloading of Na^+^ from the xylem to protect the leaf blades [54], then a Ca^2+^-dependent inhibitory effect might be a negative feature as it could prevent Na^+^ transport into stelar cells in shoots, such as in leaf sheaths. It is also possible that *HvPIP2;8* might be an entry point for Na^+^ influx in root surface cells under salt stress when there is a low external Ca^2+^. When equivalent external K^+^ and Na^+^ were available, we observed that the presence of the K^+^ inhibited the Na^+^ transport (Figure 2). This could have physiological relevance in relation to regulating monovalent ion transport in conditions where K^+^ is abundant relative to when Na^+^ is in excess, such as in saline conditions. Water and ion transport are involved in the regulation of cell expansion, and aquaporins that can transport both ions and water could also potentially have key functions in cell expansion processes [55,56]. To understand the physiological roles of *HvPIP2;8*, it will be necessary to phenotype the barley control and mutant or transgenic lines that significantly vary in the abundance of *HvPIP2;8*. It will also be essential to determine the specific location and abundance of the *HvPIP2;8* protein in both roots and shoots in control and salt-stressed conditions.

## 4. Materials and Methods

### 4.1. Plant Materials and Growth Conditions

For sterilization, the seeds of barley (*Hordeum vulgare* L., cv. Haruna-Nijo, cv. K305, cv. I743) were treated with 10% H_2_O_2_ for 10 min. After 1 day, seeds were immersed in distilled water with aeration, and the germinated seeds were transplanted and hydroponically cultured with aeration in 3.5 L pots with 0.25 mM CaSO_4_ for 2 days, and then for more days after replacing the medium with the hydroponic solution as described previously [57]. With aeration, all pots were in the dark for first 1 day, and then for a 12 h dark/12 h light cycle with fluorescent lamps of 150 photons µmol m^−2^ s^−1^ in an airconditioned room (23 ± 0.5 °C). Salt stress (100 mM or 200 mM NaCl) was treated with 5-day-old seedlings by adding 20.5 g and 40.9 g NaCl to the 3.5 L of hydroponic solution, respectively.

### 4.2. Extraction of RNA and Gene Expression Analysis by RT-PCR and Real-Time Quantitative PCR (qPCR)

Shoots and roots were sampled from hydroponically grown barley plants at 5, 6, or 10 days old (control), and 1 or 5 days after the treatment of 100 mM or 200 mM NaCl. Samples were rinsed and immediately frozen in liquid nitrogen. Total RNA was extracted using a mortar and pestle and the RNeasy Plant Mini Kit (Qiagen, Hilden, Germany). cDNA was synthesized using the Rever Tra Ace kit (Toyobo, Osaka Japan). cDNA fragments of *HvPIP2;8* (GenBank accession number AK356299) and Elongation factor 1α (*EF1α*, GenBank accession number Z50789) as the internal control were amplified with a set of specific primers (Supplementary Table S1). Absolute quantification was performed in the qPCR analysis using the 7300 real-time PCR machine (Applied Biosystem, Foster City, CA, USA) with PCR conditions of 50 °C for 2 min, 95 °C for 10 min, 33 cycles of 95 °C for 15 s, and 58 °C for 1 min to analyze the expression level of *HvPIP2;8*. Transcript copy numbers were quantified from three technical replications, and two biological independent experiments were conducted.

### 4.3. Preparation of HvPIP cRNAs

The coding region of each *HvPIP* (from *Hordeum vulgare* cv. Haruna-Nijo) was cloned into the vector pXβG-ev1 [24,31]. Each construct was linearized and cRNAs were synthesized using the mMESSAGE mMACHINE T3 kit (Ambion, Austin, TX, USA), with a final concentration of 1 µg/µL.

### 4.4. Expression of HvPIPs in X. laevis Oocytes

Oocytes were obtained from adult female *X. laevis* frogs and placed in a modified Barth’s solution (MBS: 88 mM NaCl, 1 mM KCl, 2.4 mM NaHCO_3_, 1.5 mM Tris-HCl (pH 7.6), 0.3 mM Ca(NO_3_)_2_^.^4H_2_O, 0.41 mM CaCl_2_^.^4H_2_O, 0.82 mM MgSO_4_^.^7H_2_O, 10 µg mL^−1^ penicillin sodium salt, and 10 µg mL^−1^ streptomycin sulfate) or in a low Na^+^ Ringer solution (62 mM NaCl, 36 mM KCl, 5 mM MgCl_2_, 0.6 mM CaCl_2_, 5 mM HEPES (pH 7.6), 5% (*v*/*v*) horse serum, and antibiotics (0.05 mg mL^−1^ tetracycline, 100 units mL^−1^ penicillin, and 0.1 mg mL^−1^ streptomycin)) in the experiments comparing *HvPIP2;8* S285D relative to *HvPIP2;8* WT. The lobes were torn apart and treated with 1 mg mL^−1^ collagenase B (type B, Boehringer Mannheim, Germany) in Ca-free MBS for 1.5 h. Isolated oocytes were washed several times and incubated in MBS for 1 day at 20 °C before the microinjection.

Oocytes were injected with 10 ng of *HvPIP2* cRNA. As for *HvPIP1s*, 40 ng of each cRNA was injected. Oocytes were injected with nuclear-free water as a negative control in all experiments. Injected oocytes were incubated for 24 h to 48 h at 20 °C in MBS or a low Na^+^ Ringer solution until the electrophysiological experiments were performed. The experiments using frog oocytes were approved by the Animal Care and Use Committee, Okayama University (approval number OKU-2017271), which follows the related international and domestic regulations. Experiments testing *HvPIP2;8* wild type (WT) relative to the *HvPIP2;8* S285D mutant, and additional replication experiments confirming the HvPIP2 relative ionic conductance, were performed at the University of Adelaide, Waite Research Institute. For the experiments comparing *HvPIP2;8* WT an *HvPIP2;8* S285D, the currents were recorded in “Na100”: 100 mM NaCl; 2 mM KCl, 1 mM MgCl_2_, 5 mM HEPES, 50 μM CaCl_2_, 100 mM NaCl or 100 mM KCl, osmolality 220 mosmol Kg^−1^ (adjusted with D-mannitol), and a pH of 8.5. Ionic conductance was calculated by taking the slope of a regression of the linear region across the reversal potential (−40 mV to +20 mV). Oocytes were either untreated or were pre-treated in a low Na^+^ Ringer solution that contained 10 µM H7 dihydrochloride (H7) for 2 h before TEVC and the swelling assay.

### 4.5. Electrophysiology

Two-electrode voltage clamp (TEVC) was performed using *X. laevis* oocytes injected with water or cRNA. Borosilicate glass pipettes (Harvard Apparatus, GC150TF-10, 1.5 mm O.D. × 1.17 mm I.D.) for voltage and current injecting electrodes were pulled and filled with 3 M KCl. A bath clamp system was used to minimize the effect of series resistance in the bath solution. The bath current and voltage sensing electrodes consisted of a silver–silver chloride electrode connected to the bath by 3% agar with 3 M KCl bridges. All bath solutions contained a background of high external calcium concentration (1.8 mM MgCl_2_, 1.8 mM Mannitol, 1.8 mM CaCl_2_, 10 mM HEPES, pH 7.5 with Tris) or low external calcium concentration (1.8 mM MgCl_2_, 1.8 mM EGTA (ethylene glycol-bis (β-aminoethyl ether)-*N,N,N′,N′*-tetraacetic acid), 1.8 mM CaCl_2_, 10 mM HEPES, pH 7.5 with Tris), except where otherwise mentioned. Osmolality of the bath solutions was adjusted to 200 mosmol Kg^−1^ with supplemental mannitol. Free Ca^2+^ was calculated using https://somapp.ucdmc.ucdavis.edu/pharmacology/bers/maxchelator/CaMgATPEGTA-NIST-Plot.htm. Divalent cations (Ca^2+^, Mg^2+^, Ba^2+^ and Cd^2+^) and monovalent cations (Na^+^, K^+^, Li^+^, Cs^+^ and Rb^+^) were added as chloride salts or gluconate salts. Each oocyte was carefully pierced with the voltage and current electrodes and the membrane voltage was allowed to stabilize. Conductance responses were monitored through the experiments by the repeat of steps from −120 mV to +30 mV with 2 s steady states and 5 s intervals. The recording was performed and analyzed with an Axoclamp 900A amplifier and Clampex 9.0 software (Molecular Devices, CA, USA) at room temperature (20–22 °C).

For the analysis of the *HvPIP2;8* 285S mutants relative to the *HvPIP2;8* wild type (WT), the oocyte preparation, oocyte water permeability, and electrophysiology have been described [14]. The water- or cRNA-injected oocytes were incubated in a low Na^+^ Ringer solution (62 mM NaCl, 36 mM KCl, 5 mM MgCl_2_, 0.6 mM CaCl_2_, 5 mM HEPES, 5% (*v*/*v*) horse serum, and antibiotics (0.05mg mL^−1^ tetracycline, 100 units mL^−1^ penicillin/0.1 mg mL^−1^ streptomycin), pH 7.6, for 24–36 h. The water- or cRNA-injected oocytes were pre-incubated in a 3 mL iso-osmotic solution (5 mM NaCl, 2 mM KCl, 1 mM MgCl_2_, 50 µM CaCl_2_, pH 8.5) with an osmolality of 240 mosmol Kg^−1^ (adjusted with D-mannitol) for 1 h prior to being transferred to a solution with the same ionic composition (5 mM NaCl, 2 mM KCl, 1 mM MgCl_2_, 50 µM CaCl_2_, pH 8.5), with an osmolality of 45 mosmol Kg^−1^ for the photometric swelling assay. Two-electrode voltage clamp (TEVC) recordings were performed on *X. laevis* oocytes 24–36 h post injection. Preparation of glass pipettes was as described [14]. TEVC experiments were performed using an Oocyte Clamp OC-725C (Warner Instruments, Hamden, CT, USA) with a Digidata 1440A data acquisition system interface (Axon Instruments, Foster City, CA, USA). Injected oocytes were continuously perfused with solution after being pierced with the voltage and current electrodes and allowed to stabilize. TEVC was performed in solutions consisting of 100 mM NaCl (“Na100”) or 100 mM KCl (“K100”) in a basal solution (2 mM KCl, 1 mM MgCl_2_, and 5 mM HEPES, osmolality was adjusted to 220–230 mosmol Kg^−1^ with D-mannitol) with 50 µM CaCl_2_ and a pH of 8.5. For experiments involving kinase inhibitor H7, the injected oocytes were incubated prior to TEVC in a low Na^+^ Ringer solution (described previously) supplemented with 10 µM H7 dihydrochloride (Sigma, #17016) from concentrated stocks dissolved in water. Steady-state currents were recorded starting from a −40 mV holding potential for 0.5 s and ranging from 40 mV to −120 mV with 20 mV decrements for 0.5 s before following a −40 mV pulse for another 0.5 s. Ionic conductance was calculated by taking the slope of a regression of the linear region across the reversal potential (−40 mV to +20 mV). TEVC recordings were analyzed with CLAMPEX 9.0 software (pClamp 9.0 Molecular Devices, CA, USA).

Biological replication included testing of different oocytes from different batches harvested from different frogs, and the oocyte and batch replication was three or more; the representative result from one or more oocyte batch from each experiment is included in the figures.

### 4.6. Statistical Analysis

Statistical analysis was conducted using SPSS statistics software (version 20). Analysis of variance was identified by one-way ANOVA followed by the least significant difference (LSD) test at the 0.05 level; or one-way ANOVA followed by Fisher’s post-hoc test.

## 5. Conclusions

Our electrophysiological analyses of barley HvPIP aquaporins expressed in *X. laevis* oocytes have shown that *HvPIP2;8* facilitates an ionic conductance at the plasma membrane in the presence of Na^+^ and/or K^+^ in an external Ca^2+^-sensitive manner. Co-expression of HvPIP1s and *HvPIP2;8* significantly reduced the *HvPIP2;8*-dependent ionic conductance, and our manipulation of protein phosphorylation revealed that this channel is likely to be subject to complex regulation involving heteromerization and post-translational modification. These findings progress our insight into the potential roles of plant aquaporins under salt stress and they are likely to inspire future research to uncover the molecular and structural mechanisms that control the dual permeability of aquaporins for ions and water, and testing of the physiological role of *HvPIP2;8* in planta.

## Supplementary Materials

Supplementary materials can be found at https://www.mdpi.com/1422-0067/21/19/7135/s1. Supplementary Figure S1. The inhibition of Na^+^ transport by external free Ca^2+^ concentration in *Xenopus laevis* oocytes expressing *HvPIP2;8* in the presence of 86.4 mM NaCl and 9.6 mM KCl. The background solution contained (1.8 mM MgCl_2_, 1.8 mM EGTA, 1.8 mM CaCl_2_, 10 mM HEPES pH 7.5 with Tris) for external free 30 µM Ca^2+^ to 1 mM Ca^2+^ concentration; and (1.8 mM MgCl_2_, 1.8 mM Mannitol, 1.8 mM CaCl_2_, 10 mM HEPES pH 7.5 with Tris) for external free 1.8 mM Ca^2+^ concentration as control. Steady-state current-voltage curves of *X. laevis* oocytes injected with 10 ng of cRNA per oocyte from the same batch (n = 5–6 for *HvPIP2;8* cRNA). Supplementary Figure S2. Reversal potentials of *HvPIP2;8* mediated ionic currents in the presence of 48 mM NaCl with 48 mM each alkaline cation. Data are means ± SE (n = 4–5). Supplementary Figure S3. Interaction between K^+^ and Na^+^ on *HvPIP2;8* mediated currents. (A) Effect of external Na^+^ cation on K^+^ permeability. (B) Effect of external K^+^ cation on Na^+^ permeability through *HvPIP2;8*-transporter. The total concentration of (Na + K) was constantly 96 mM. Na^+^ and K^+^ external concentration (chloride salt) were 9 different ratios bath solutions with high calcium condition contained a background (1.8 mM MgCl_2_, 1.8 mM CaCl_2_, 1.8 mM Mannitol, 10 mM HEPES pH 7.5 with Tris). Steady-state current-voltage curves of *X. laevis* oocytes injected with 10 ng of cRNA per oocyte from the same batch. Data are means ± SE, n = 5–6. Supplementary Figure S4. (A,B) *HvPIP2;8* WT and *HvPIP2;8* S285D cRNA injected oocytes exhibited batch to batch variation in three independent frogs. Oocytes were injected with 46 nL water (Control) or with 46 nL water (n = 11) containing 23 ng *HvPIP2;8* WT (n = 30) or *HvPIP2;8* S285D (n = 40) cRNA. Ionic conductance and osmotic water permeability (P_os_) of cRNA injected oocytes was determined via TEVC and swelling assay, respectively. Dots in black indicates 1st batch, dots in red indicates 2nd batch and dots in blue indicates 3rd batch. Data is shown as mean ± SEM where each data point represents an individual oocyte. Significant differences (*p* < 0.05) are indicated by different letters (one-way ANOVA, Fisher’s post-test). (C) Kinase inhibitor H7 treatment increase Na^+^ and K^+^ conductance in *HvPIP2;8* S285D expressing oocytes. Oocytes were injected with 46 nL water (Control) or with 46 nL water containing 23 ng *HvPIP2;8* WT or *HvPIP2;8* S285D cRNA. Ionic conductance of cRNA injected oocytes was determined via the TEVC. For TEVC, currents were tested in solution containing 100 mM NaCl and 2 mM KCl or 100 mM KCl; and each solution contained: 1 mM MgCl_2_, 5 mM HEPES, 50 μM CaCl_2_, 100 mM NaCl or 100 mM KCl, osmolality of 220 mosmol Kg^−1^, pH 8.5. There was 2 mM KCl in the Na solution, but the K^+^ solution did not contain Na^+^. Oocytes injected with water (with H7, n = 5; without H7, n = 4) or *HvPIP2;8* (with H7, n = 14; without H7, n = 10) or *HvPIP2;8* S285D (with H7, n = 17; without H7, n = 11) cRNA were either untreated or were pre-treated in low Na^+^ Ringers solution that contained with 10 µM dihydrochloride (H7) for 2 h before TEVC. Data is shown as mean ± SE where each data point represents an individual oocyte. Significant differences are indicated by one asterisk (*p* < 0.05) or two asterisks (*p* < 0.01) (one-way ANOVA, Fisher’s post-test). (D) Predicted protein kinase A (PKA) and protein kinase C (PKC), phosphorylation sites in *HvPIP2;8*. Blue, amino acids predicted to be phosphorylated by PKA. Red, amino acids predicted to be phosphorylated by PKC. NetPhos 3.1 server (http://www.cbs.dtu.dk/services/NetPhos/, access time 07/2020) was used to determine the sites. (E) Predicted amino acid sequence alignment for *HvPIP2;8* and AtPIP2;1 indicating predicted phosphorylation sites based on NetPhos (http://www.cbs.dtu.dk/services/NetPhos/) analysis. Sites predicted to be phosphorylated by either PKA or PKC are listed, along with key sites of interest related to gating *Spinacia oleracea* SoPIP2;1 (Leu200) [58] and protein interactions AtPIP2;1 G103 [6]. (F) Predicted amino acid sequence alignment for eight HvPIP2s relative to AtPIP2;1; it is important to note that the sequence of HvPIPs in different barley varieties may differ. Supplementary Figure S5. Expression analysis of *HvPIP2;8* using RT-PCR. Salt tolerant K305 (A,B), salt sensitive I743 (C,D), and moderate tolerant Haruna-Nijo where *HvPIP2;8* was isolated originally (E,F) were grown 5 days without salt stress then grow more 1 day and 5 days with or without supplemented 100 or 200 mM NaCl. Total RNA was isolated from shoots and roots and *HvPIP2;8* fragments (A,C,E) or internal standard EF1α (B,D,F) fragments were amplified. Black and white arrow heads indicate PCR products of *HvPIP2;8* and EF1α, respectively. Representative result was shown in 3 replications. M, DNA size markers. Table S1. Gene-specific primer pairs used in PCR experiments. Table S2. Ionic conductance of oocytes injected HvPIP2s or water in the presence of 86.4 mM NaCl and 9.6 mM KCl. Ionic conductance was calculated based on the data obtained from V = −75 mV to −120 mV of the membrane potential in Figure 1. Data are means ± SE (n = 5–7), ns (not significant), * (*p* < 0.05) using one-way ANOVA with Duncan’s multiple comparisons test. Table S3. Reversal potential of ion currents in oocytes expressing *HvPIP2;8* in the presence of NaCl or KCl. Free external Ca^2+^ was calculated as about 30 µM in low Ca^2+^ and 1.8 mM in high Ca^2+^ solutions. Data are means ± SE.
