# Supplementary material for: A Survey of Barley PIP Aquaporin Ionic Conductance Reveals Ca2+-Sensitive HvPIP2;8 Na+ and K+ Conductance
Source: Int J Mol Sci. 2020 Sep 27;21(19):7135. doi: 10.3390/ijms21197135 (PMC7582361; doi:10.3390/ijms21197135)
Supplement: Supplementary file 1 [file ijms-21-07135-s001.pdf]

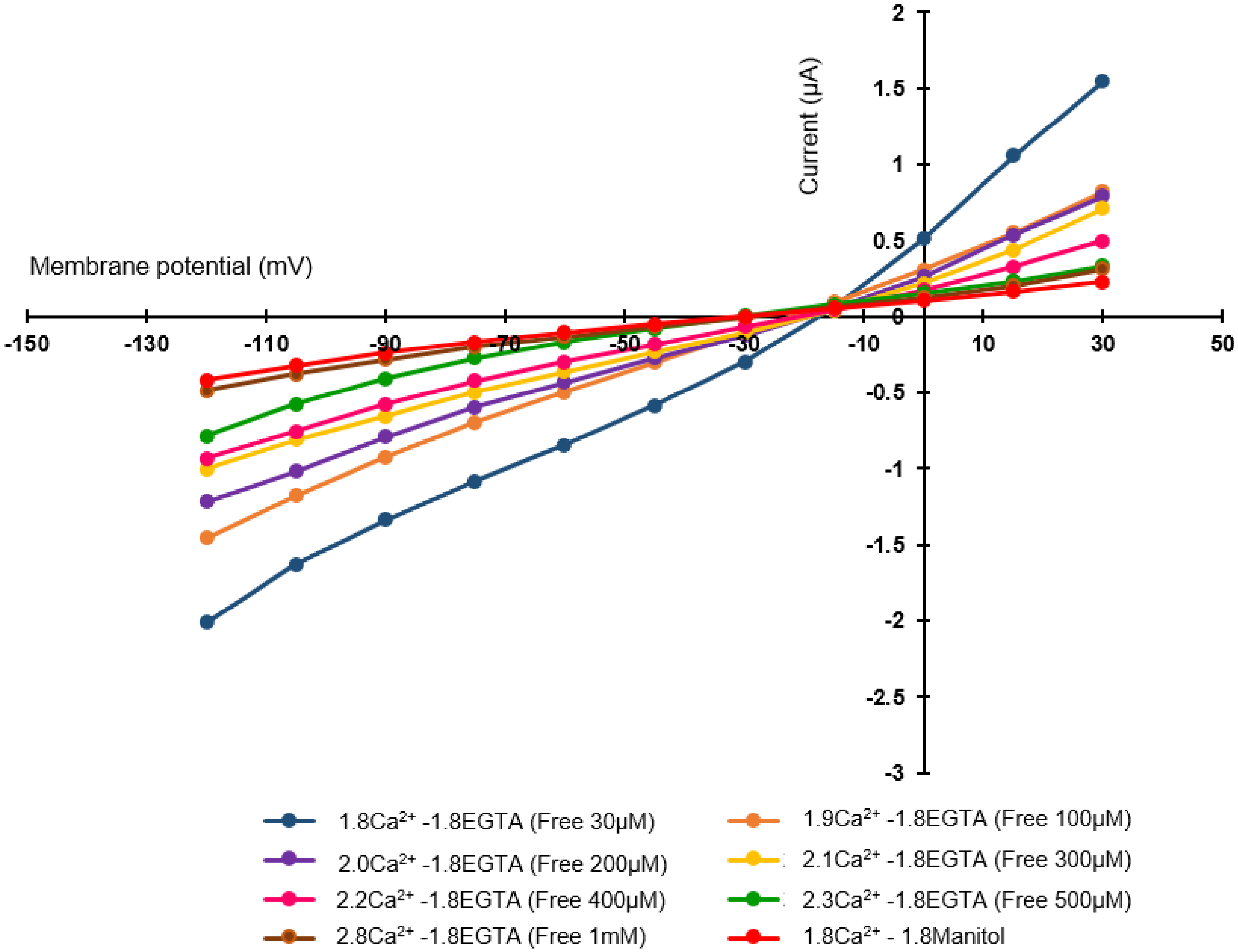

Figure S1. The inhibition of Na^+ transport by external free Ca^{2+} concentration in *Xenopus laevis* oocytes expressing *HvPIP2;8* in the presence of 86.4 mM NaCl and 9.6 mM KCl. The background solution contained (1.8 mM $MgCl_2$, 1.8 mM EGTA, 1.8 mM $CaCl_2$, 10 mM HEPES pH 7.5 with Tris) for external free 30 µM Ca^{2+} to 1 mM Ca^{2+} concentration; and (1.8 mM $MgCl_2$, 1.8 mM Mannitol, 1.8 mM $CaCl_2$, 10 mM HEPES pH 7.5 with Tris) for external free 1.8 mM Ca^{2+} concentration as control. Steady-state current-voltage curves of *X.laevis* oocytes injected with 10 ng of cRNA per oocyte from the same batch (n = 5 - 6 for *HvPIP2;8* cRNA).

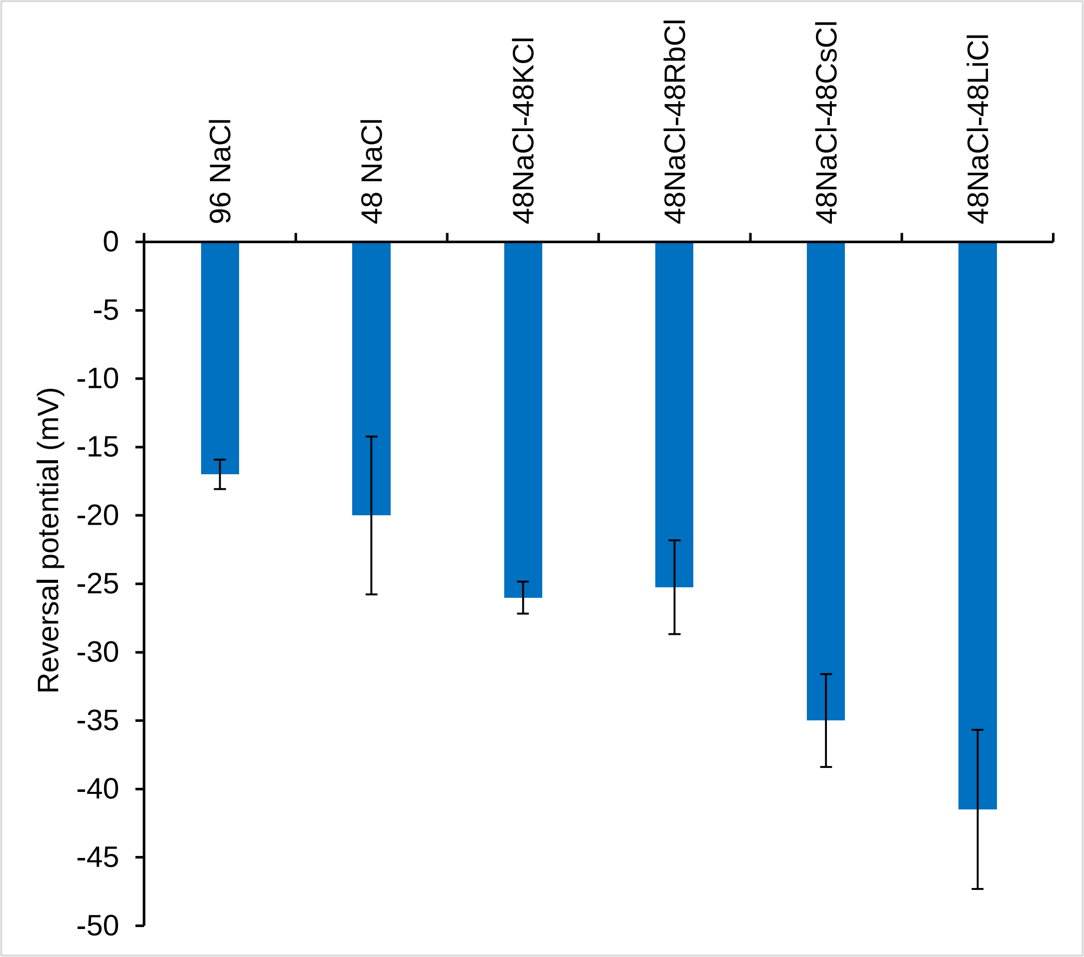

Figure S2. Reversal potentials of HvPIP2;8 mediated ionic currents in the presence of 48 mM NaCl with 48 mM each alkaline cation. Data are means ± SE (n = 4 - 5).

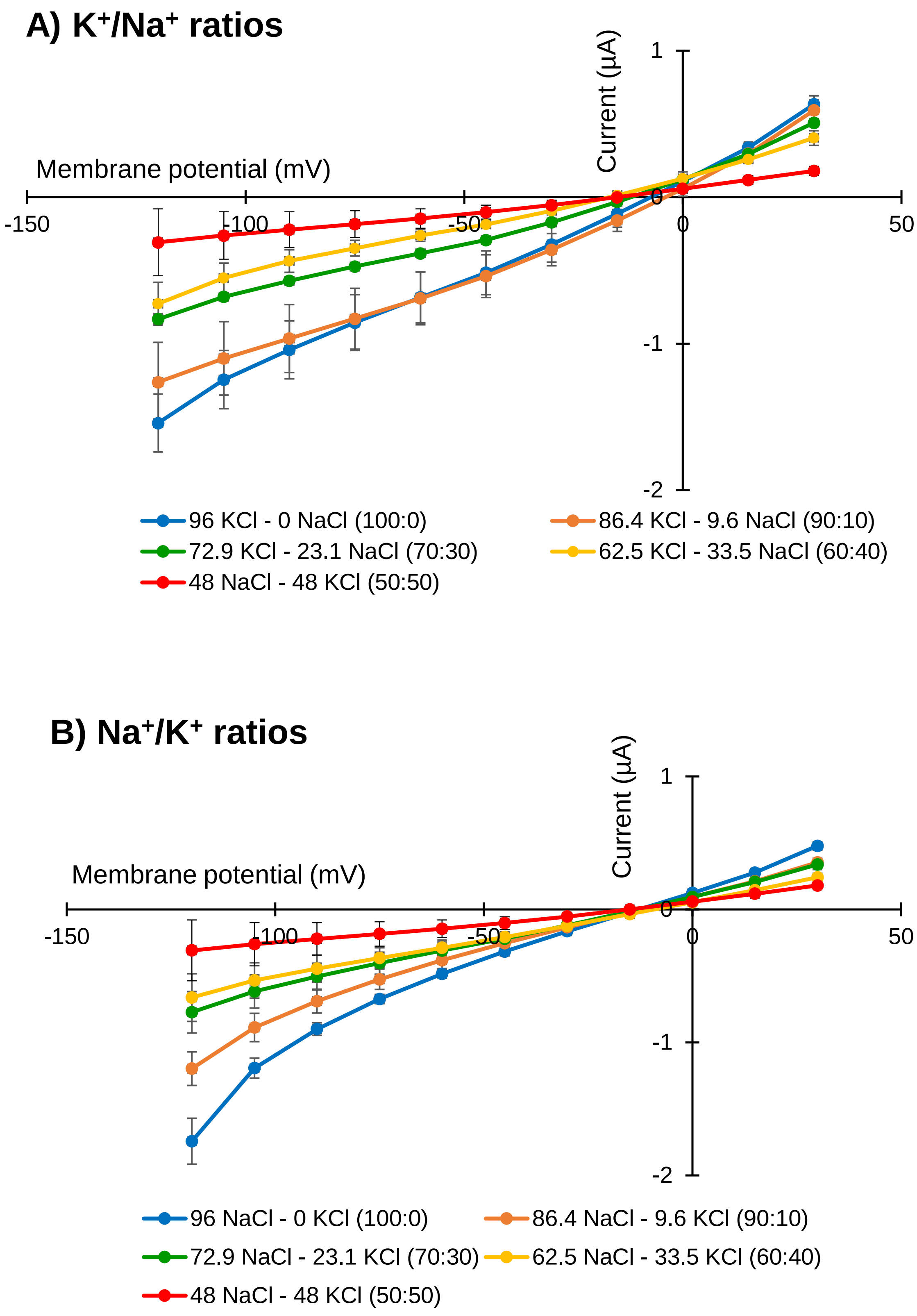

Figure S3. Interaction between K^+ and Na^+ on HvPIP2;8 mediated currents. **A)** Effect of external Na^+ cation on K^+ permeability. **B)** Effect of external K^+ cation on Na^+ permeability through HvPIP2;8-transporter. The total concentration of (Na + K) was constantly 96 mM. Na^+ and K^+ external concentration (chloride salt) were 9 different ratios bath solutions with high calcium condition contained a background (1.8 mM $MgCl_2$, 1.8 mM $CaCl_2$, 1.8 mM Mannitol, 10mM HEPES pH 7.5 with Tris). Steady-state current-voltage curves of *X.laevis* oocytes injected with 10 ng of cRNA per oocyte from the same batch. Data are means ± SE, n = 5 - 6.

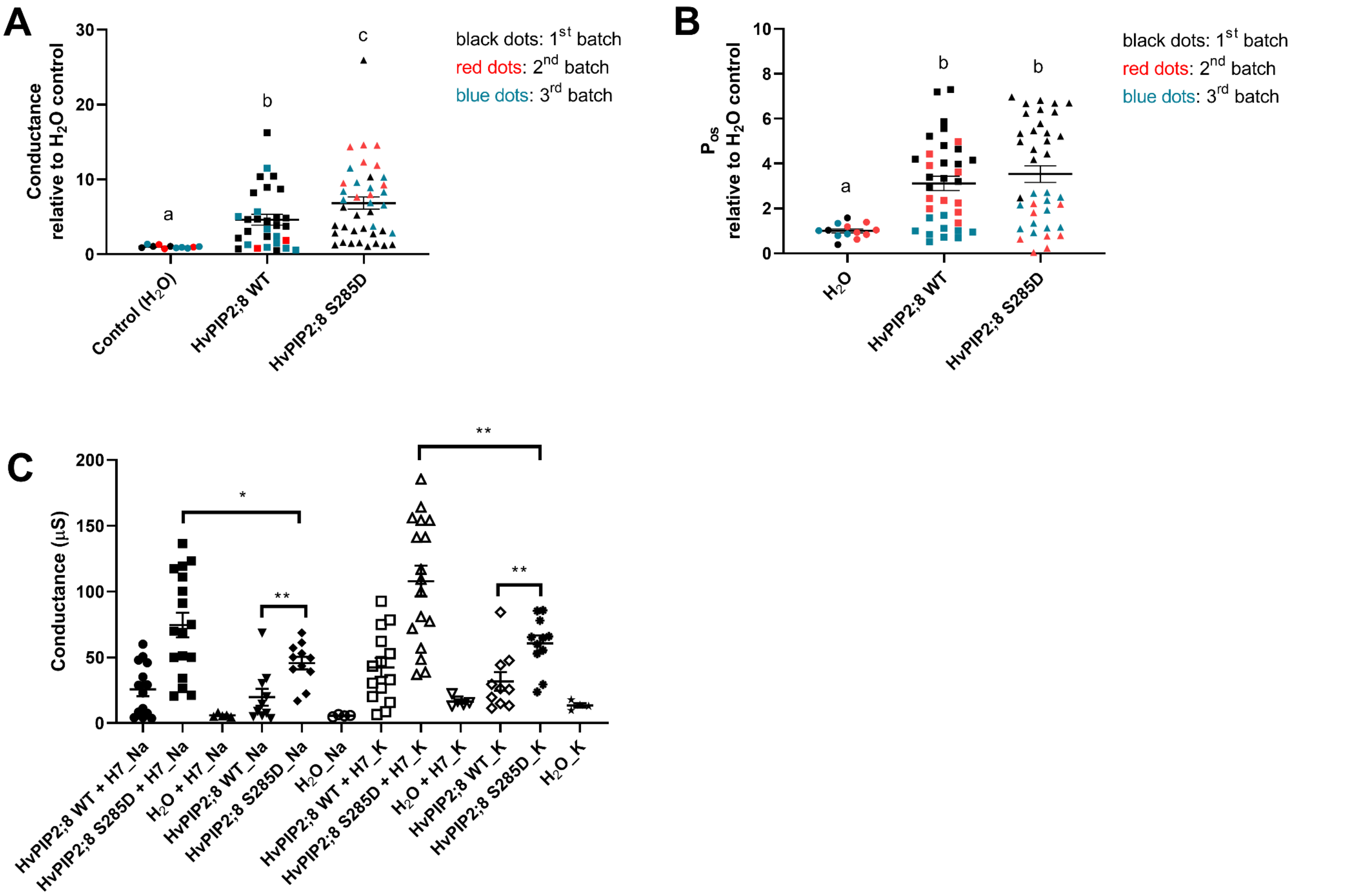
A
Conductance relative to H_2O control
30
20
10
0
a
b
c
Control (H_2O)
HvPIP2;8 WT
HvPIP2;8 S285D
black dots: 1st batch
red dots: 2nd batch
blue dots: 3rd batch
B
P_{os} relative to H_2O control
10
8
6
4
2
0
a
b
b
H_2O
HvPIP2;8 WT
HvPIP2;8 S285D
black dots: 1st batch
red dots: 2nd batch
blue dots: 3rd batch
C
Conductance (μS)
200
150
100
50
0
*
**
**
**
HvPIP2;8 WT + H7_Na
HvPIP2;8 S285D + H7_Na
H_2O + H7_Na
HvPIP2;8 WT_Na
HvPIP2;8 S285D_Na
H_2O_Na
HvPIP2;8 WT + H7_K
HvPIP2;8 S285D + H7_K
H_2O + H7_K
HvPIP2;8 WT_K
HvPIP2;8 S285D_K
H_2O_K

D

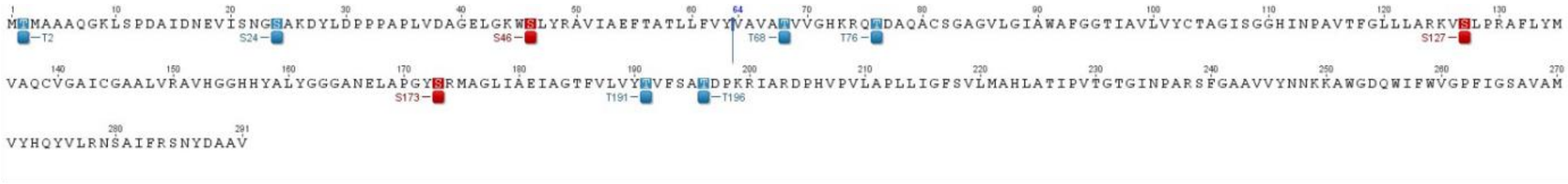

- Predicted PKA phosphorylation site
- Predicted PKC phosphorylation site

E

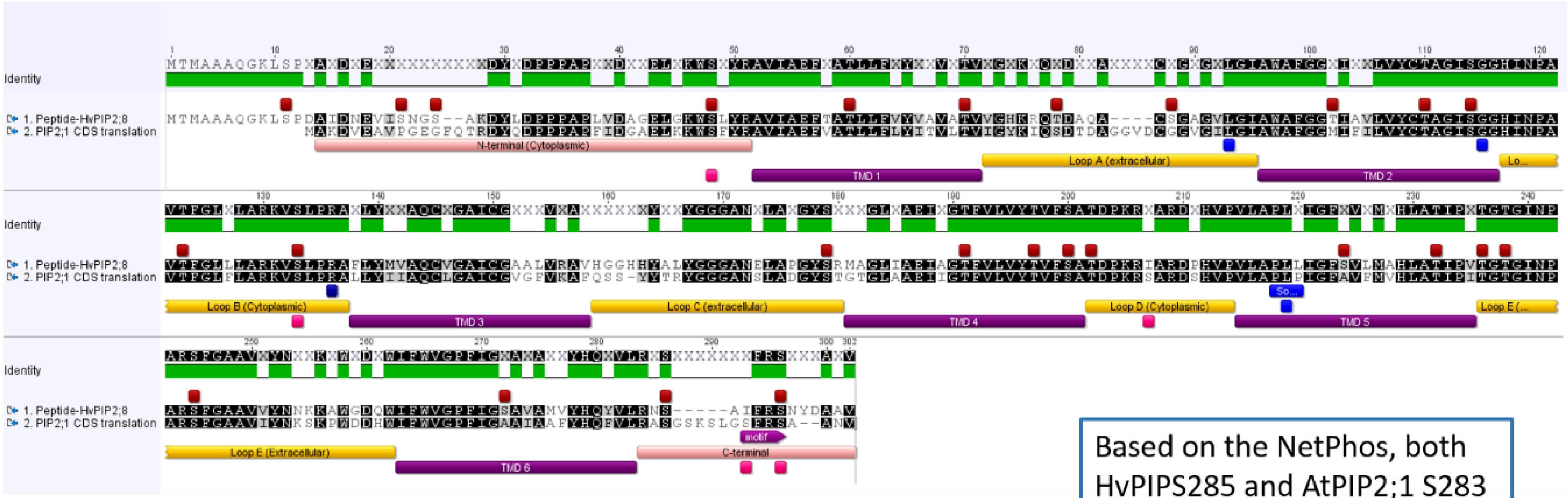

- Predicted P sites in HvPIP2;8
- Published P sites in AtPIP2;1
- SoPIP2;1 Leu 200 and G103(AtPIP2;1)

Based on the NetPhos, both HvPIPS285 and AtPIP2;1 S283 are not be able to be phosphorylated by PKA and PKC.

NetPhos 3.1 results:
AtPIP2;1 P sites
By PKA: S36, S121, S198, S273
By PKC: T58, T169, T184, T189, T219, S273, S280
http://www.cbs.dtu.dk/cgi-bin/webface2.fcgi?jobid=5EE84189000031D73375E5FE&wait=20

NetPhos 3.1 results:
HvPIP2;8 P sites
By PKA: S46 (AtPIP2;1 S36), S127 (AtPIP2;1S121), S173
By PKC: T2, S24, T68, T76, T191, T196
http://www.cbs.dtu.dk/cgi-bin/webface2.fcgi?jobid=5EE84286000031D7795C84C5&wait=20

F

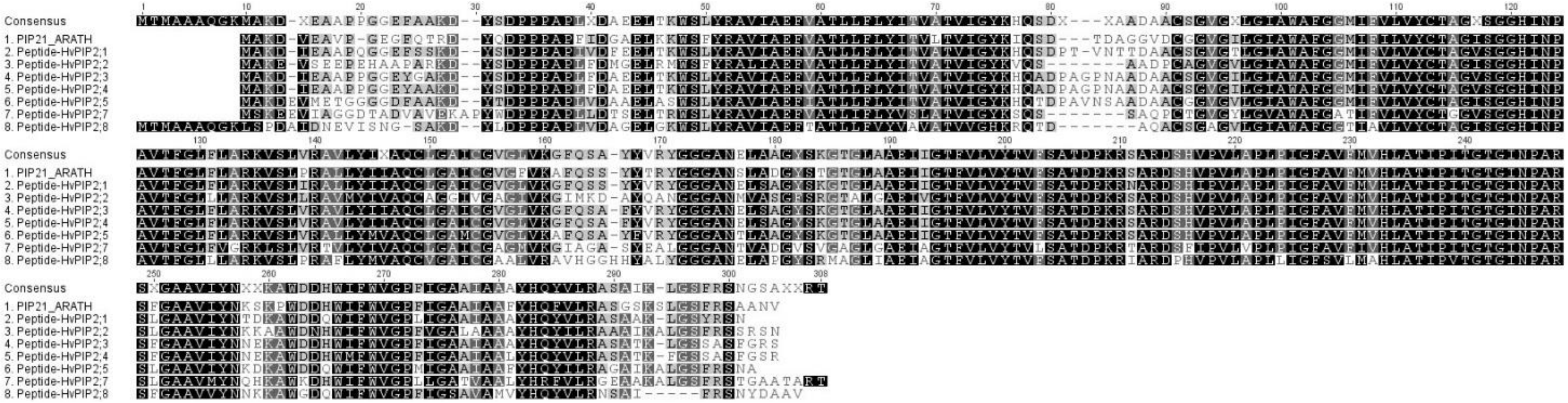

Supplementary Figure S4.

Supplementary Figure S4. (A-B) HvPIP2;8WT and HvPIP2;8S285D cRNA injected oocytes exhibited batch to batch variation in three independent frogs. Oocytes were injected with 46 nL water (Control) or with 46 nL water (n=11) containing 23 ng HvPIP2;8 WT (n=30) or HvPIP2;8 S285D (n=40) cRNA. Ionic conductance and osmotic water permeability (Pos) of cRNA injected oocytes was determined via TEVC and swelling assay, respectively. Dots in black indicates 1st batch, dots in red indicates 2nd batch and dots in blue indicates 3rd batch. Data is shown as mean ± SEM where each data point represents an individual oocyte. Significant differences (P<0.05) are indicated by different letters (one-way ANOVA, Fisher's post-test). (C) Kinase inhibitor H7 treatment increase Na^+ and K^+ conductance in HvPIP2;8 S285D expressing oocytes. Oocytes were injected with 46 nL water (Control) or with 46 nL water containing 23 ng HvPIP2;8 WT or HvPIP2;8 S285D cRNA. Ionic conductance of cRNA injected oocytes was determined via the TEVC. For TEVC, currents were tested in solution containing 100 mM NaCl and 2mM KCl or 100 mM KCl; and each solution contained: 1 mM $MgCl_2$, 5 mM HEPES, 50 µM $CaCl_2$, 100 mM NaCl or 100 mM KCl, osmolality of 220 mosmol.kg^{-1}, pH 8.5. There was 2 mM KCl in the Na solution, but the K^+ solution did not contain Na^+. Oocytes injected with water (with H7,n= 5; without H7, n= 4) or HvPIP2;8 (with H7, n=14; without H7, n=10) or HvPIP2;8 S285D (with H7, n= 17; without H7, n=11) cRNA were either untreated or were pre-treated in low Na^+ Ringers solution that contained with 10 µM dihydrochloride (H7) for 2h before TEVC. Data is shown as mean ± SE where each data point represents an individual oocyte. Significant differences are indicated by one asterisk (P<0.05) or two asterisks (P<0.01) (one-way ANOVA, Fisher's post-test). (D) Predicted protein kinase A (PKA) and protein kinase C (PKC), phosphorylation sites in HvPIP2;8. Blue, amino acids predicted to be phosphorylated by PKA. Red, amino acids predicted to be phosphorylated by PKC. NetPhos 3.1 server

(http://www.cbs.dtu.dk/services/NetPhos/, access time 07/2020) was used to determine the sites. (E) Predicted amino acid sequence alignment for HvPIP2;8 and AtPIP2;1 indicating predicted phosphorylation sites based on NetPhos (http://www.cbs.dtu.dk/services/NetPhos/) analysis. Sites predicted to be phosphorylated by either PKA or PKC are listed, along with key sites of interest related to gating *Spinacia oleracea* SoPIP2;1 (Leu200) [58] and protein interactions AtPIP2;1 G103 [59]. (F) Predicted amino acid sequence alignment for eight HvPIP2s relative to AtPIP2;1; it is important to note that the sequence of HvPIPs in different barley varieties may differ

Materials and methods

Oocyte preparation, oocytes water permeability and electrophysiology were described in Qiu et al., 2020.

Water or cRNA injected oocytes were incubated in low Na^+ Ringer's solution (62 mM NaCl, 36 mM KCl, 5 mM $MgCl_2$, 0.6 mM $CaCl_2$, 5 mM HEPES, 5% (v/v) horse serum and antibiotics (0.05mg mL-1 tetracycline, 100 units mL-1 penicillin/0.1 mg mL-1 streptomycin)), pH 7.6 for 24-36 h.

Water or CRNA injected oocytes were pre-incubated in 3 mL iso-osmotic solution (5 mM NaCl, 2 mM KCl, 1 mM MgCl2, 50 µM CaCl2, pH 8.5) with an osmolality of 240 mosmol Kg^{-1} (adjusted with D-mannitol) for 1 h prior to being transferred to a solution with the same ionic composition (5 mM NaCl, 2 mM KCl, 1 mM MgCl2, 50 µM CaCl2, pH 8.5) with an osmolality of 45 mosmol Kkg^{-1} for the photometric swelling assay.

Two-electrode voltage clamp (TEVC) recordings were performed on *X. laevis* oocytes 24-36 h post injection. Preparation of glass pipettes was as described in Byrt et al., (2017). TEVC experiments were performed using an Oocyte Clamp OC-725C (Warner Instruments, Hamden, CT, USA) with a Digidata 1440A data acquisition system interface (Axon Instruments, Foster City, CA, USA). Injected oocytes were continuously perfused with solution after being pierced with the voltage and current electrodes and allowed to stabilise. TEVC was performed in solutions consisting of 100 mM NaCl ('Na100') or 100 mM KCl ('K100') in a basal solution (2 mM KCl, 1 mM $MgCl_2$ and 5 mM HEPES, osmolality was adjusted to 220-230 mosmol Kg^{-1} with D-mannitol) with 50 µM $CaCl_2$ and pH 8.5. For experiments involving kinase inhibitor H7, injected oocytes were incubated prior to TEVC in Low Na^+ Ringers (described previously) supplemented with 10 µM H7 dihydrochloride (Sigma, #17016) from concentrated stocks dissolved in water. Steady–state currents were recorded starting from –40 mV holding potential for 0.5 s and ranging from 40 mV to –120 mV with 20 mV decrements for 0.5 s before following a –40 mV pulse for another 0.5 s. Ionic conductance was calculated by taking the slope of a regression of the linear region across the reversal potential (–40 mV to +20 mV). TEVC recordings were analysed with CLAMPEX 9.0 software (pClamp 9.0 Molecular Devices, CA, USA).

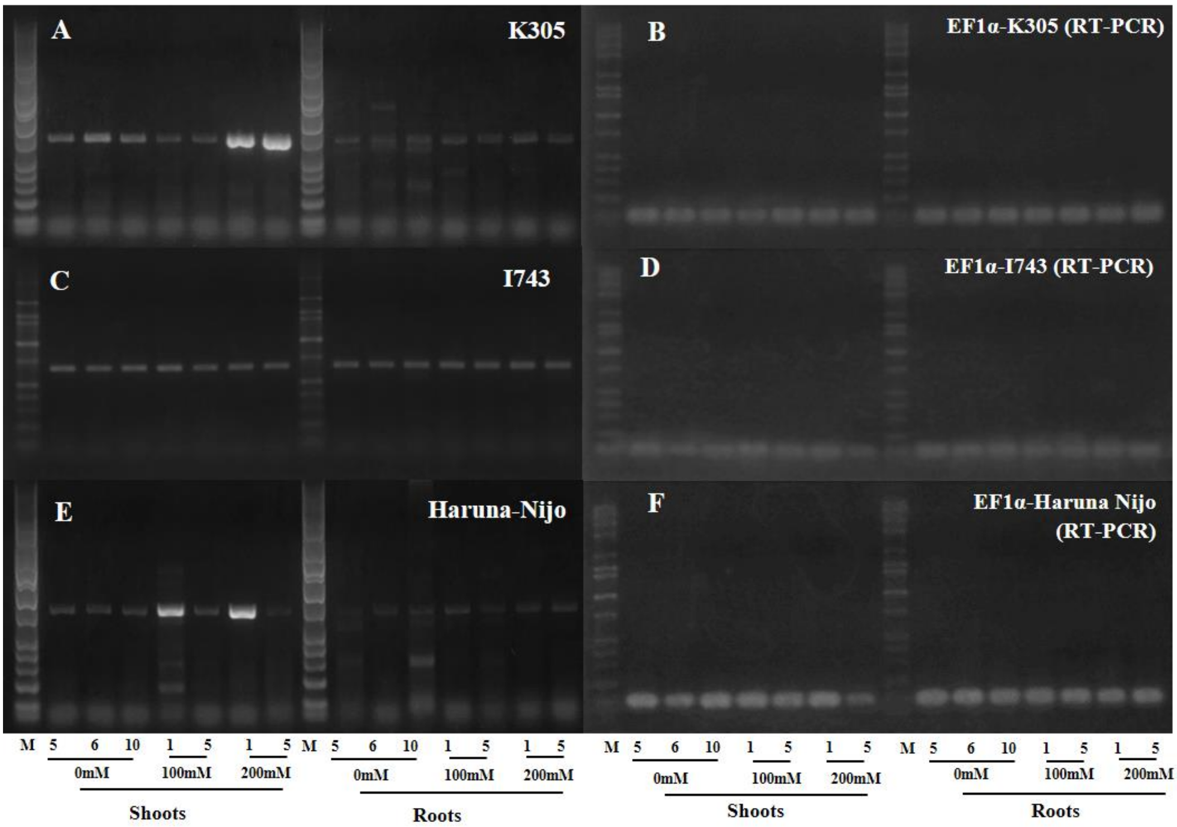

Figure S5. Expression analysis of HvPIP2;8 using RT-PCR. Salt tolerant K305 (A, B), salt sensitive I743 (C, D), and Haruna-Nijo where HvPIP2;8 was isolated originally (E, F) were grown 5 days without salt stress then grow more 1 day and 5 days with or without supplemented 100 or 200 mM NaCl. Total RNA was isolated from shoots and roots and HvPIP2;8 fragments (A, C, E) or internal standard EF1α (B, D, F) fragments were amplified. Representative result was shown in 3 replications. M, DNA size marker.

Table S1. Gene-specific primer pairs used in PCR experiments.

Gene name	Forward primer (5′-3′)	Reverse primer (5′-3′)
HvPIP2;8 (full length)	GGAGATCTAGCTTCATGACTATGGCCG	CCAGATCTAAGTAGCTAGACGGCG
HvPIP2;8 (RT-PCR)	ACACAAGCGCCAGACCGACG	GCCAGGTTGATGCTACGGCGG
HvPIP2;8 (qPCR)	TTGGGGAGACCAGTGGATCT	GCCAGGTTGATGCTACGGCGG
HvEF1α	GATAGTTGTTTTAGTCGCTTGGGTTATT	CACCAACACAACCGAACGATAC

Table S2. Ionic conductance of oocytes injected HvPIP2s or water in the presence of 86.4 mM NaCl and 9.6 mM KCl.

Ionic conductance was calculated based on the data obtained from V = -75 mV to -120 mV of the membrane potential in Figure 1. Data are means ± SE (n = 4 - 7), ns (not significant), * ($P < 0.05$) using one-way ANOVA with Duncan's multiple comparisons test.

Ionic conductance (μS)	Water (control)	HvPIP2s						
		2;1	2;2	2;3	2;4	2;5	2;7	2;8
Low Ca^{2+} (30 μM)	3.3 ± 0.6^{ns}	8.3 ± 2.1^{ns}	5.5 ± 0.4^{ns}	5.30 ± 0.8^{ns}	6.7 ± 1.5^{ns}	4.8 ± 0.6^{ns}	4.1 ± 0.8^{ns}	$36.5 \pm 5.5^{*}$
High Ca^{2+} (1.8 mM)	3.4 ± 2.4^{ns}	3.4 ± 0.4^{ns}	2.4 ± 1.4^{ns}	3.6 ± 0.6^{ns}	4.5 ± 0.6^{ns}	3.2 ± 2.3^{ns}	3.8 ± 0.8^{ns}	$14.5 \pm 4.5^{*}$

Table S3. Reversal potential of ion currents in oocytes expressing HvPIP2;8 in the presence of NaCl or KCl.

Free external Ca^{2+} was calculated as about 30 µM in low Ca^{2+} and 1.8 mM in high Ca^{2+} solutions. Data are means ± SE.

Reversal potential (mV)	86.4 mM NaCl + 9.6 mM KCl	86.4 mM KCl + 9.6 mM NaCl
Low Ca^{2+}	-10.30 ± 1.10	-12.33 ± 1.05
High Ca^{2+}	-10.60 ± 1.02	-12.21 ± 0.97
